# Sphingosine-1-Phosphate Receptor 4 links neutrophils and early local inflammation to lymphocyte recruitment into the draining lymph node to facilitate robust germinal center formation

**DOI:** 10.3389/fimmu.2024.1427509

**Published:** 2024-08-12

**Authors:** Andrea J. Luker, Abigail Wukitch, Joseph M. Kulinski, Sundar Ganesan, Juraj Kabat, Justin Lack, Pamela Frischmeyer-Guerrerio, Dean D. Metcalfe, Ana Olivera

**Affiliations:** ^1^ Laboratory of Allergic Diseases, National Institute of Allergy and Infectious Diseases (NIAID), National Institutes of Health (NIH), Bethesda, MD, United States; ^2^ Biological Imaging Section, Collaborative Research Technologies Branch (CRT), NIAID, NIH, Bethesda, MD, United States; ^3^ Integrated Data Sciences Section (IDSS), Research Technologies Branch (RTB), NIAID, NIH, Bethesda, MD, United States

**Keywords:** Sphingosine-1-phosphate receptor 4, lymph node hypertrophy, germinal center reactions, inflammation, neutrophils, innate cells

## Abstract

The successful development of germinal centers (GC) relies heavily on innate mechanisms to amplify the initial inflammatory cascade. In addition to their role in antigen presentation, innate cells are essential for the redirection of circulating lymphocytes toward the draining lymph node (dLN) to maximize antigen surveillance. Sphingosine-1-Phosphate (S1P) and its receptors (S1PR1-5) affect various aspects of immunity; however, the role of S1PR4 in regulating an immune response is not well understood. Here we use a footpad model of localized T_H_1 inflammation to carefully monitor changes in leukocyte populations within the blood, the immunized tissue, and the dLN. Within hours of immunization, neutrophils failed to adequately mobilize and infiltrate into the footpad tissue of S1PR4^-/-^ mice, thereby diminishing the local vascular changes thought to be necessary for redirecting circulating cells toward the inflamed region. Neutrophil depletion with anti-Ly6G antibodies significantly reduced early tissue edema as well as the redirection and initial accumulation of naïve lymphocytes in dLN of WT mice, while the effects were less prominent or absent in S1PR4^-/-^ dLN. Adoptive transfer experiments further demonstrated that the lymphocyte homing deficiencies *in vivo* were not intrinsic to the donor S1PR4^-/-^ lymphocytes, but were instead attributed to differences within the S1PR4-deficient host. Reduced cell recruitment in S1PR4^-/-^ mice would seed the dLN with fewer antigen-respondent lymphocytes and indeed, dLN hypertrophy at the peak of the immune response was severely diminished, with attenuated GC and activation pathways in these mice. Histological examination of the S1PR4^-/-^ dLN also revealed an underdeveloped vascular network with reduced expression of the leukocyte tethering ligand, PNAd, within high endothelial venule regions, suggesting inadequate growth of the dLN meant to support a robust GC response. Thus, our study reveals that S1PR4 may link early immune modulation by neutrophils to the initial recruitment of circulating lymphocytes and downstream expansion and maturation of the dLN, thereby contributing to optimal GC development during an adaptive response.

## Introduction

Sphingosine-1-phosphate (S1P) is a pleiotropic lipid mediator that binds to a family of five G protein-coupled receptors (S1PR1–5), whose expression varies widely among tissue type. Signaling by these receptors orchestrates a number of physiological processes in various organ systems, including the immune system (1–4). For example, S1PR1 is most widely known for its critical role in lymphocyte egress from secondary lymphoid organs (2, 5, 6), but it has also been shown to promote naïve lymphocyte survival (7) and influence regulatory T (T_reg_) cell differentiation (8, 9). Within the germinal center (GC), differential regulation of both S1PR1 and S1PR2 is vital for the retention and interaction of effector cells to allow proper formation of T follicular helper (T_FH_) and GC B cells (10–12). S1PR3 and S1PR5 have been reported to regulate the maturation and localization of dendritic cells (DCs) (13), monocytes (14), and NK cells (15). Of particular interest, S1PR4 is uniquely limited to the hematopoietic compartment where it is highly expressed on many myeloid (16–18) and lymphoid cells (19–22). While S1PR4 is thought to be involved in both innate and adaptive responses (23, 24), its specific immunological functions remain poorly understood.

Among several functional studies on S1PR4 knockout (S1PR4^-/-^) B and T cells, there are reports of certain lymphocyte-intrinsic roles for S1PR4 in regulating B1 B cell egress, T cell migration into afferent lymphatics, and proliferation and survival of CD8^+^ T cells (20, 22, 25, 26). However, accumulating evidence also suggests that S1PR4-mediated activation of myeloid cells dictates the downstream immunomodulatory effects (25, 27–29). For example, S1PR4-deficiency affects DC migration and cytokine production, thereby impacting T cell function and differentiation (27, 30). Thus, two different airway sensitization models in S1PR4-deficient mice found aggravated pulmonary inflammation driven by increased recruitment and activation of granulocytes (27, 29). In contrast, another study found that loss of S1PR4 signaling reduced psoriasis-like skin inflammation by impairing chemokine and cytokine production by macrophages, thus attenuating recruitment of monocytes (28). Two additional studies also suggested that S1PR4 may affect neutrophil homeostasis and trafficking, although these investigations were conducted in mice with existing chronic inflammation (16), or severe and lethal abnormalities due to the deletion of the major S1P regulatory enzyme, S1P lyase 1 (31). Interestingly, one large-scale meta-analysis study identified a rare missense variant in *S1PR4* that was associated with lower circulating neutrophil counts across multiple patient cohorts (32). Overall, the immunomodulatory effects of S1PR4 seem to depend on the specific cell populations involved and the environmental cues within a particular model. Importantly, however, it appears that S1PR4 regulation of innate cells, particularly of myeloid lineage, is vital for the propagation of pro- or anti-inflammatory cascades.

While early components of innate immunity induce drastic changes within the local tissue, the effects of this acute inflammation also impact the draining lymph node (dLN) to prime the adaptive immune response. Activated innate cells and the soluble factors they release thus alter the microenvironment to enhance circulation and lymphocyte recruitment toward the dLN, thereby heavily impacting the success of the downstream GC reactions (33, 34). Consistent with these observations, a recent study described reduced GC size in the spleens of S1PR4^-/-^ mice challenged with systemic infection (23). With the potential roles of S1PR4 still poorly understood, we sought to investigate in S1PR-deficient mice some of the initial events leading up to GC formation using a well-characterized T_H_1 footpad immunization model. This approach limits the confounding factors associated with chronic or live systemic infection models, allowing our analysis to focus on localized tissue inflammation and the direct downstream development of GC within the dLN. We found that dLN hypertrophy, driven by the recruitment of naïve lymphocytes, was severely impaired in S1PR4^-/-^ mice. While this was followed by the formation of fewer and smaller GCs, our studies revealed minimal lymphocyte-intrinsic abnormalities. Instead, the loss of S1PR4 caused a disruption in innate events soon after immunization, particularly involving neutrophil mobilization, which in turn impaired proper seeding of lymphocytes into the dLN. Further, attenuated environmental cues and cellular infiltration dampened the feed-forward mechanisms that drive the vascular remodeling required to support a robust adaptive immune response. Our study thus expands the knowledge on the immune-modulatory role of S1PR4 in the development of GC reactions and reveals that S1PR4 may link neutrophil mobilization and early inflammation to proper lymphocyte recruitment into dLN and effective GC formation.

## Materials and methods

### Mice and immunizations

*S1pr4^−/−^* mice and *S1pr4^+/+^* mice (referred to as wildtype, WT) were originally obtained from The Jackson Laboratory (Bar Harbor, Maine) (strain B6.129P2-*S1pr4tm1Dgen*/J) and subsequently backcrossed to C57/BL6 at least 10 times (26). The S1PR4 colony was maintained within a Taconic Biosciences barrier facility (Germantown, NY). Mice were transferred two weeks prior to the initiation of the experiments to a vivarium of the National Institute of Allergy and Infectious Diseases (NIAID), accredited by the American Association for the Accreditation of Laboratory Animal Care (AAALAC). The protocols with live mice were performed under an animal study proposal (LAD2E) approved by the NIAID Division of Intramural Research (DIR) Animal Care and Use Committee under the guidance of the Office of Animal Care and Use of the National Institutes of Health. Male and female mice, aged between 8-12 weeks old, were used for experiments with WT littermates serving as controls. Sedation was induced using an anesthesia chamber delivering Isoflurane and oxygen-enhanced air. Euthanasia was achieved via overdose of inhaled Isoflurane followed by a secondary physical method.

Anesthetized mice were immunized subcutaneously in the hind footpad using a 26- or 30-gauge needle with 20 µL of a Freund’s Incomplete Adjuvant (IFA) emulsion (InvivoGen) containing 50 µg ovalbumin (OVA, Biosearch Technologies) and 5 µg lipopolysaccharide (LPS, E. coli O111:B4, MilliporeSigma) to induce a robust T_H_1 immune response. The emulsion was prepared as previously described (35). Briefly, a solution of 0.5 mg/mL LPS and 5 mg/mL OVA in sterile PBS was combined 1:1 with IFA by passing between two borosilicate glass syringes affixed to a three-way stopcock until a stable emulsion was achieved. Animals were euthanized at various time-points after immunization, and the draining popliteal lymph nodes (dLN) collected for further processing. In some experiments, the non-draining contralateral LNs were used as a control.

The S1PR4-specific antagonist, CYM50358-hydrochloride (R&D Systems), was resuspended into a 100 mM stock with sterile water and diluted with Kolliphor EL (MilliporeSigma) 1:10 (Kolliphor EL: CYM50358). A working solution of 2 mg/mL of CYM50358 containing 0.5% Kolliphor EL in PBS was used to inject mice i.v. with 10 mg/kg CYM50358 or an equivalent volume of vehicle 30 min prior to footpad injection. For neutrophil depletion experiments, mice were injected *i.p.* with 200 µg of *InVivo*Mab anti-mouse Ly6G (clone 1A8) or *InVivo*Mab rat IgG2a, κ isotype control (clone 2A3; Bio X Cell) prepared in sterile PBS on two days prior and on the day of footpad immunization.

### Subcutaneous footpad flush

Anesthetized mice were injected in both hind footpads with 20 µL of IFA/LPS/OVA 3 hrs prior to euthanasia. Paws were removed above the ankle where the tissue was secured using a hemostat and the toes were excised to create an opening for collection. The footpads were flushed with 2 mL of a buffer containing 0.2% bovine serum albumin (BSA) and <0.1% Sodium Azide injected subcutaneously with a 22G needle. The cell infiltrates flushed from both paws of a single mouse were pooled to obtain enough cells for analysis. The samples were thoroughly washed to remove the residual emulsion and the resulting pellet was briefly treated with ACK Lysis Buffer before performing a cell count and staining for flow cytometry.

### Immunofluorescence and immunohistochemistry

For the staining of the LN vasculature, dLNs were cleaned of fat connective tissue and fixed for 24 hrs in 4% paraformaldehyde and dehydrated in a 30% sucrose solution overnight, before controlled freezing in Tissue-Tek O.C.T. compound (Sakura) on a metal block partially submerged in an ethanol/dry ice slurry. Tissue sections (10 µm) of the lymph nodes were prepared by Histoserv, Inc. (Germantown, MD). Frozen sections were rehydrated in PBS and blocked with a solution containing 1% BSA, 2% normal mouse serum, 2% normal rat serum, 2% Fc block, and 0.3% Triton X-100 for 1 hr at room temperature. Primary antibodies were added and incubated overnight in a humidity chamber at 4°C (see **Supplementary Table 1**). Slides were counterstained with NucBlue Fixed Cell ReadyProbes Reagent (DAPI, Invitrogen) then coverslipped with Prolong Diamond Antifade Mountant (Invitrogen).

For triplex staining of CD4^+^ T cells, B cells, and GC B cells, LNs were fixed for 24 hrs in 10% neutral-buffered formalin, then paraffin-embedded (FFPE). IHC staining on 5 µm sections was performed on the Leica Biosystems Bond RX autostainer using the Bond Polymer Refine Kit (Leica Biosystems DS9800). After antigen retrieval with EDTA (Bond Epitope Retrieval 2), sections were incubated for 30 min with anti-CD4, followed by ImmPRESS HRP-conjugated Goat anti-Rat (Vector Labs) and OPAL Fluorophore 570 (Akoya Biosciences). The CD4 antibody complex was stripped by heating with Bond Epitope Retrieval 2, followed by Bond Epitope Retrieval 1. Sections were then incubated with anti-CD45R/B220 for 60 min, followed by ImmPRESS HRP-conjugated Goat anti-Rat and Alexa Fluor 647 AffiniPure Goat anti-HRP for 30 min. Finally, sections were probed with biotin-conjugated peanut agglutinin (PNA) for 60 min to stain for GC B cells, followed by Alexa Fluor 488-conjugated streptavidin for 30 min. See **Supplementary Table 1** for details on the antibodies. Omission of primary antibodies was used for the negative control. Slides were removed from the Bond autostainer, stained with DAPI, and coverslipped with Prolong Gold antifade reagent (Invitrogen).

### RNAscope *in situ* hybridization

The expression of *S1pr4* and *S1pr1* was detected by staining 5 µm FFPE LN sections with the RNAscope 2.5 LS Mm-S1pr4-C1 and Mm-S1pr1-C2 (ACD Bio) probes with the RNAscope LS Multiplex Fluorescent Assay (ACD Bio), using the Bond RX auto-stainer (Leica Biosystems) with a tissue pretreatment of 30 min at 100°C with Bond Epitope Retrieval Solution 2 (Leica Biosystems) and 1:750 dilution of OPAL 570 or OPAL 690 reagents (Akoya Biosciences), respectively. IHC for CD4^+^ T cells was performed after RNAscope by incubating with anti-mouse CD4 antibody for 30 min and secondary antibody Rabbit anti-Rat IgG (Vector Laboratories) using the Bond Polymer Refine Kit (Leica Biosystems) minus Post Primary reagent, DAB, and Hematoxylin, with OPAL 520 reagent (1:750) for 30 min. The CD4 antibody was stripped away with Bond Epitope Retrieval Solution 1 for 20 min at 95°C. B cells were detected by subsequently staining with an anti-mouse CD45R/B220 antibody for 30 min and secondary antibody Rabbit anti-Rat IgG (Vector Laboratories) using the Bond Polymer Refine Kit (Leica Biosystems) as above with OPAL 620 reagent (1:750) for 30 min. See **Supplementary Table 1** for details on the primary antibodies. The RNAscope 3-plex LS Multiplex Negative Control Probe (*dapB* gene in channels C1, C2, and C3) followed by IHC with no primary antibodies was used as a negative control. The RNAscope LS 2.5 3-plex Positive Control Probe-Mm was used as a technical control to ensure the RNA quality of tissue sections was suitable for staining.

### Confocal imaging

Confocal images were collected using a Leica DMI6000 Sp8 WLL confocal microscope (Leica Microsystems, Exton, PA) enabled with 20X NA 0.7 or 40X NA 1.32 oil immersion objective. Images were acquired using a highly sensitive Hybrid detector with constant laser intensity and detector gain to maintain the consistency to compare the differences within and between different tissues. Images of several tiles or entire tissues were collected using automated tiling method using LASX-Navigator module to observe a global perspective of the tissue of interest.

Acquired images were further analyzed or processed using Imaris image processing software (Bitplane USA, South Windsor, CT) or QuPath-0.4.3 open-source software (36).

For lumen vessel diameter measurements, the longest region of a fully enclosed vessel was identified, and the perpendicular measurement was recorded as the diameter. Multiple measurements (between 24-108) were collected from each section, then a value was assigned for each individual LN by averaging the top 20 largest measurements.

For Region of Interest studies, GC masks were defined as areas of PNA^+^ staining and other histological features using surface rendering and masking tools of Imaris software package (version 10.1) image processing software. The data was then imported into FlowJo for population analysis.

### Whole mount brightfield histology

Hind paws were prepared similarly to a previous report (37). Briefly, the tissues were fixed in 10% neutral-buffered formalin for 24-72 hrs followed by decalcification, sectioning, and H&E staining performed by Histoserv, Inc. Brightfield images were collected using a NanoZoomer S60 (Hamamatsu) digital slide scanner with 40X objective. Acquired images were further visualized and analyzed using NDP.view2 software (Hamamatsu).

### Preparation of single-cell suspensions

In most cases, LN or spleens were mechanically dissociated and filtered into a single-cell suspension. For DC analysis, dLN and spleens were first digested for 15-30 min at 37°C with 2 mg/mL collagenase D (Roche) or 25 µg/mL Liberase-TM (Roche), respectively, in the presence of 50 µg/mL DNase1. White blood cells were enriched from heparinized whole blood by incubating for 1-3 min in 20 mL ice-cold ACK lysis buffer. Splenocytes were also subjected to ACK lysis to eliminate red blood cells. All samples were thoroughly washed and filtered through 40 µm cell strainers. Absolute counts and viability were determined using acridine orange/propidium iodide stain on a LUNA-FL Automated Cell Counter (Logos Biosystems) or via CountBright Absolute Counting Beads (Invitrogen) during flow cytometric data collection.

### Flow cytometry and cell sorting

Freshly prepared single-cell suspensions were simultaneously stained with Zombie Aqua (Biolegend) fixable viability dye and Fc block in PBS for 15 min at room temperature. Incubation with antibodies against G protein-coupled receptors (i.e. CXCR5) was also performed at room temperature for the final 10 minutes in PBS to improve staining. All remaining incubations with antibody cocktails for relevant cell surface markers were performed for 15 min at 4°C in Brilliant Stain Buffer Plus (BD Biosciences) followed by fixation with BD Cytofix (BD Biosciences) also for 15 min at 4°C (see **Supplementary Table 2** for details on flow antibodies and **Supplementary Figure 1** for examples of immune cell gating strategies within different tissues). For intracellular cytokine staining, cells were stored in Cyto-Last buffer (Biolegend) prior to being permeabilized using 1X Intracellular Staining Permeabilization Wash Buffer (Biolegend) according to manufacturer’s instructions. For intracellular antibody detection, the cell surface was saturated with unlabeled IgG, then permeabilized and stained for the presence of intracellular Ig. For trans-nuclear staining of transcription factors or Ki67, cells were prepared using the True-Nuclear Transcription Factor Buffer Set (Biolegend). In some cases, whole blood was stained then incubated with 1X RBC Lysis/Fixation Solution (Biolegend) according to manufacturer’s instructions. Data acquisition was performed with an LSRFortessa Cell Analyzer (BD Biosciences) and analysis using FlowJo (v10) software.

GC B cells and T_FH_ cells were sorted from dLN of immunized mice (n=6 WT and n=6 S1PR4^-/-^) using a FACSAria II (BD Biosciences) for subsequent RNA-Seq analysis as described below. T cells were sorted as T_FH_ (TCRβ^+^ CD4^+^ PD1^+^ CXCR5^+^) or non-T_FH_ (TCRβ^+^ CD4^+^ PD1^-^ CXCR5^-^) on Day 6 and B cells as GC (B220^+^ GL7^+^ Fas^+^) or non-GC (B220^+^ GL7^-^ Fas^-^) on Day 9. Due to the staggered kinetics or emergence of these effector populations, the time-points of Day 6 and Day 9 were chosen to correspond with the initial peak of T_FH_ cells and GC B cells, respectively.

### RNA-Seq of sorted cell populations

Freshly sorted cell populations were resuspended and stored in RNAprotect Cell Reagent (Qiagen) and subsequently RNA was isolated using a RNeasy Micro Kit (Qiagen). RNA quality and purity was confirmed with a 2100 Bioanalyzer (Agilent). Total RNA for standard (>100 ng) or low-input (>6 ng) from each sample of the sorted populations was used for an mRNA capture with oligo-dT coated magnetic beads. The mRNA was fragmented, and then a random-primed cDNA synthesis was performed and paired-end libraries were generated using the Illumina Stranded Total RNA Prep, Ligation with Ribo-Zero Plus. The resulting double-stranded libraries were pooled and sequenced on a Novaseq 6000 S1 flow cell. The samples had 63 to 155 million pass filter reads with more than 92% of bases above the quality score of Q30. Samples were processed from raw fastq through to raw expression values using the RNA-seek workflow (https://github.com/OpenOmics/RNA-seek). Within that workflow, raw reads were trimmed for adapters and low-quality bases using Cutadapt v2.10 (38) before alignment to the mouse reference genome (mm10) and the Gencode Release M30 annotation using STAR v2.5.3 in two-pass mode (39). PCR duplicates were marked using Picard MarkDuplicates v2.27.3 (https://broadinstitute.github.io/picard/). The average mapping rate of all samples was 82-90%, with 65-75% of reads mapping to coding exons (sequencing and mapping statistics are given in **Supplementary Table 3**). From the alignment files, gene-level quantification was performed using RSEM v1.3.0 (40) and pairwise differential expression was performed using DESeq2 (41) implemented in iDEP v94 (42). Raw and normalized expression matrices were uploaded to the Gene Expression Omnibus (GEO) (43) and are available under the accession number GSE266438 (https://www.ncbi.nlm.nih.gov/geo/query/acc.cgi?acc=GSE266438). Pathway enrichment was performed through the use of QIAGEN IPA (QIAGEN Inc., https://digitalinsights.qiagen.com/IPA) (44).

### Naïve B cell culture and activation

Naïve B cells from spleens were isolated by negative selection using CD43-depletion according to manufacturer’s (Miltenyi) instructions to achieve a purity >95%. Naïve B cells were cultured in round-bottom 96-well plates in complete RPMI supplemented with 10% FBS, 25 mM HEPES (Corning), 1X MEM nonessential amino acids (Corning), 1X pen/strep, 1mM Sodium Pyruvate (MilliporeSigma), 50 µM 2-mercaptoethanol, 1 µg/mL purified hamster anti-mouse CD40 (HM40-3, Biolegend) and 10 ng/mL recombinant mouse IL-4 (Peprotech) or 1 µg/mL LPS (from E. coli O111:B4, MilliporeSigma). Cells were seeded at 10,000 or 100,000 cells per well when activated with anti-CD40/IL-4 or anti-CD40/LPS, respectively.

### Naïve T cell culture and activation

Naïve CD4^+^ T cells were purified and expanded using a Naïve CD4^+^ T cell Isolation Kit and T Cell Activation/Expansion Kit while under the skewing conditions provided in the CytoBox T_H_1 or CytoBox T_H_2 kits (all from Miltentyi Biotech). Some T cells were cultured with IL-2 only (T_H_0). After five days, successful skewing was confirmed by trans-nuclear staining of transcription factors, then cells were activated with PMA (50 ng/mL) and Ionomycin (1 µg/mL) in the presence of GolgiPlug (BD Biosciences) to evaluate intracellular cytokine production.

### Miles assay (Evan’s Blue dye extravasation)

Mice were injected *i.p.* with 10 µL of 0.5% Evan’s Blue dye per gram of body weight 3 hrs post-immunization and euthanized 3 hrs later (6 hrs post-immunization). Both the injected and contralateral paws were then collected at the ankle. The dye extravasated into the tissue was extracted by incubating the chopped paws in 2.5 mL of formamide per gram of tissue weight overnight at 55°C. The amount of dye in the supernatants (100 µL) was measured on a spectrophotometer at 610 nm against a formamide-only blank.

### Adoptive transfer experiments

WT and S1PR4^-/-^ naïve splenocytes isolated as described above were stained with CellTrace Yellow or CellTrace Far Red (Invitrogen) according to the manufacturer’s instructions. To ensure valid results, the colors were alternated between experimental repeats. Mixed at a 1:1 (WT:S1PR4^-/-^) ratio, 10^7^ cells were injected retro-orbitally into WT and S1PR4^-/-^ hosts that had received footpad immunizations six days prior. Draining and contralateral LNs were harvested 24 hrs later, and single cell suspensions evaluated by flow cytometry for the presence of transferred cells.

### Statistical analysis

GraphPad Prism (version 9.5.1) was used to determine statistical significance, defined as *p<0.05; **p<0.01; ***p<0.001; ****p<0.0001, using unpaired t-tests or a Two Way ANOVA with multiple comparisons. Each value represents an individual mouse (a minimum of three) or measurement, and bars represent mean and standard deviation (SD), unless otherwise indicated in the Figure Legend. Each experiment was repeated at least three independent times unless otherwise indicated in the Figure Legend.

## Results

### S1PR4-deficiency results in diminished LN expansion and an attenuated T_H_1 GC reaction

A recent publication reported reduced splenic GC formation in S1PR4^-/-^ mice following colon ascendens stent peritonitis (CASP) (23). While this is a clinically relevant model, the continuous exposure to intestinal microbes creates an environment of chronic inflammation that can confound intricate mechanistic studies. To better characterize the kinetics of GC formation in S1PR4^-/-^ mice, we chose a robust, well-defined, and localized model of T_H_1 inflammation that allows precise and timely analysis of the initial responses from the time of immunization.

**Figure 1:**
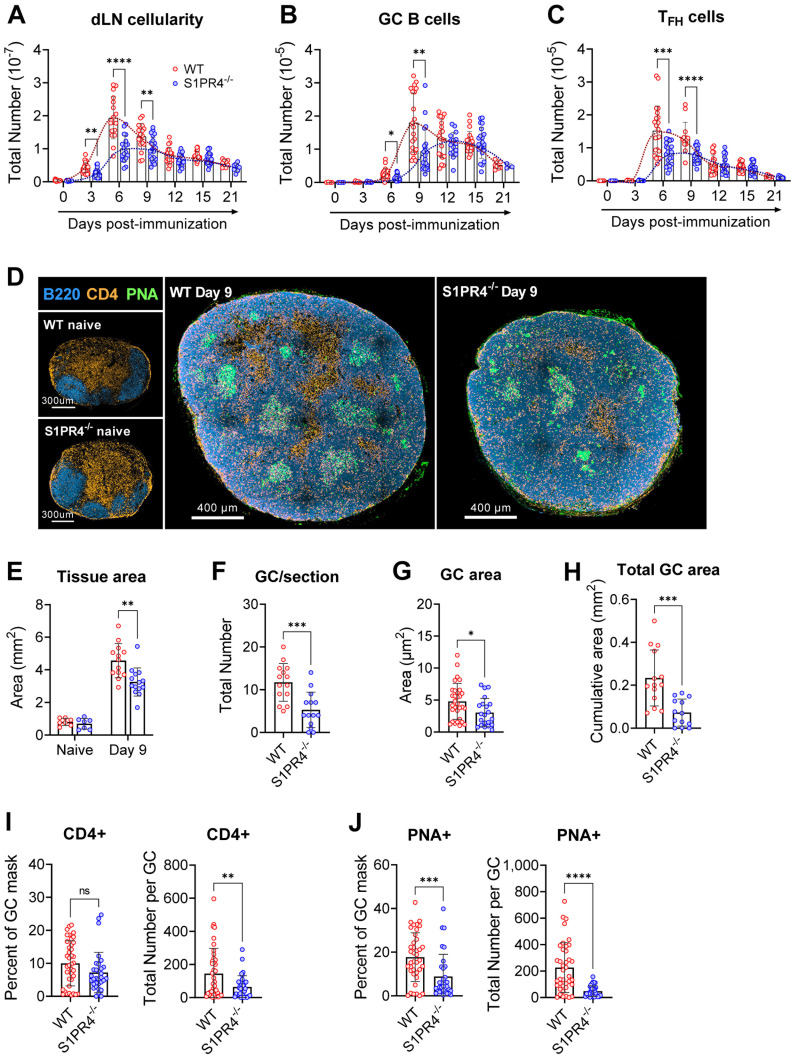
S1PR4^-/-^ mice develop an attenuated GC response with diminished histological features. dLN from WT and S1PR4^-/-^ mice were collected at various time-points after immunization with IFA/LPS/OVA in the hind footpad and analyzed for **(A)** total cellularity, **(B)** total number of GC B cells, and **(C)** total number of T_FH_ cells. The dotted lines indicate approximated curves for the kinetics of GC responses in WT (red) and S1PR4^-/-^ mice (blue). **(D)** Representative confocal images of WT and S1PR4^-/-^ dLN of naïve (left; scale bar, 300 µm) or Day 9 post-immunized (right; scale bar, 400 µm) mice showing B cells (B220 in blue), T cells (CD4 in orange), and GC (PNA in green). Histological quantification of **(E)** total dLN area of tissue section from naïve and immunized mice, **(F)** number of GC regions per LN section, **(G)** individual GC area measurements, and **(H)** cumulative area of all GC per LN section. Region of Interest studies were performed within masked GC regions and analyzed for the percent and total number of **(I)** CD4+ and **(J)** PNA^+^ cells. Red and blue circles indicate WT and S1PR4^-/-^, respectively. Values represent LN from one individual mouse **(E, F, H)** or multiple GC measurements within WT or S1PR4^-/-^ LN **(G, I, J)** from a total of 7-14 sections. Data represent Mean ± SD; ns, not statistically significant; *p<0.05; **p<0.01; ***p<0.001; ****p<0.0001 using unpaired t-tests **(A-D)** or multiple unpaired t-tests **(A-C)**.

Following subcutaneous footpad injection with an IFA emulsion containing LPS and OVA, we collected the draining popliteal lymph nodes at various time-points to monitor the reaction. In WT mice, this immunization model elicited a marked expansion of the dLN, with the total number of cells in the dLN reaching a sharp peak at Day 6 before resolving toward a more maintained, yet elevated level at Day 21 (**Figure 1A**). However, dLN expansion in S1PR4^-/-^ mice was diminished by nearly 50% at the peak of the response (Day 6) and over time did not reach the level of hypertrophy seen in WT mice. Beyond Day 9, as the responses were slowly involuting, the cellularity was comparable between the genotypes. A line approximating the kinetics of each time-course highlights the stark contrast between the bell-shaped vs attenuated plateau of WT and S1PR4^-/-^ curves, respectively (**Figure 1A**, dotted lines). Differential analysis revealed a similar reduction in the total number of both B and T cells (**Supplementary Figure 2A**). Importantly, the total number of GC B cells and T_FH_ cells were significantly reduced at the peak of the responses (**Figures 1B, C**). Despite the distinct phenotype, we found no differences in any individual population frequency that would suggest a dominant role for S1PR4 in a specific cell type (**Supplementary Figures 2B, C**).

Given this attenuated response, we next examined naïve and Day 9 dLN for structural or architectural abnormalities *in situ*. In agreement with our assessment of naive tissues (**Supplementary Table 4**), resting naïve popliteal LNs from WT and S1PR4^-/-^ mice were of similar size and organized into appropriate B and T cell compartments (**Figure 1D**, left, and **E**). Nine days post-immunization, however, S1PR4^-/-^ dLN were overall reduced in size (**Figures 1D**, right, and **E**) and had formed significantly fewer (**Figure 1F**) and smaller (**Figure 1G**) GCs, resulting in a 60-70% reduction in the cumulative GC area per dLN section (**Figure 1H**).

Furthermore, each GC contained fewer PNA^+^ B cells and CD4^+^ T cells (interpreted as T_FH_) (**Figures 1I, J**) than WT mice, suggesting S1PR4^-/-^ GCs are less densely populated with appropriately differentiated and/or activated effector cells. Of note, the frequency of GC B cells occupying the defined GC regions, but not that of CD4^+^ T cells, was reduced by 50% in S1PR4^-/-^ dLN (**Figures 1I, J**). Together, these data show that in the absence of S1PR4, the kinetics and magnitude of dLN responses and the formation of appropriate GCs are significantly altered.

Changes in expression of S1PRs in activated lymphoid tissues are thought to be important for proper retention of cells in the GC, as well as their stimulation and differentiation (6, 12, 45). However, little is known about the expression of S1PR4 during an immune response. Thus, we examined the levels of *S1pr4* in dLN 9 days post-immunization using RNAscope and RNA-sequencing of selected cell populations. RNAscope *in situ* hybridization of WT dLN sections revealed very high expression of both *S1pr4* and *S1pr1* mRNAs throughout the activated dLN, except the GC regions which were nearly devoid of these mRNA transcripts (**Supplementary 3A**), as has been reported for *S1pr1* (11, 12). This expression pattern was confirmed through next-gen sequencing of sorted populations from our activated dLN (**Supplementary 3B-C**). The deliberate downregulation of *S1pr4* mRNA within GC populations may suggest a role for S1PR4 in the proper activation, localization, or differentiation of effector lymphocytes within dLN.

### Loss of S1PR4 reduces activation pathways of effector lymphocytes during the immune response

**Figure 2:**
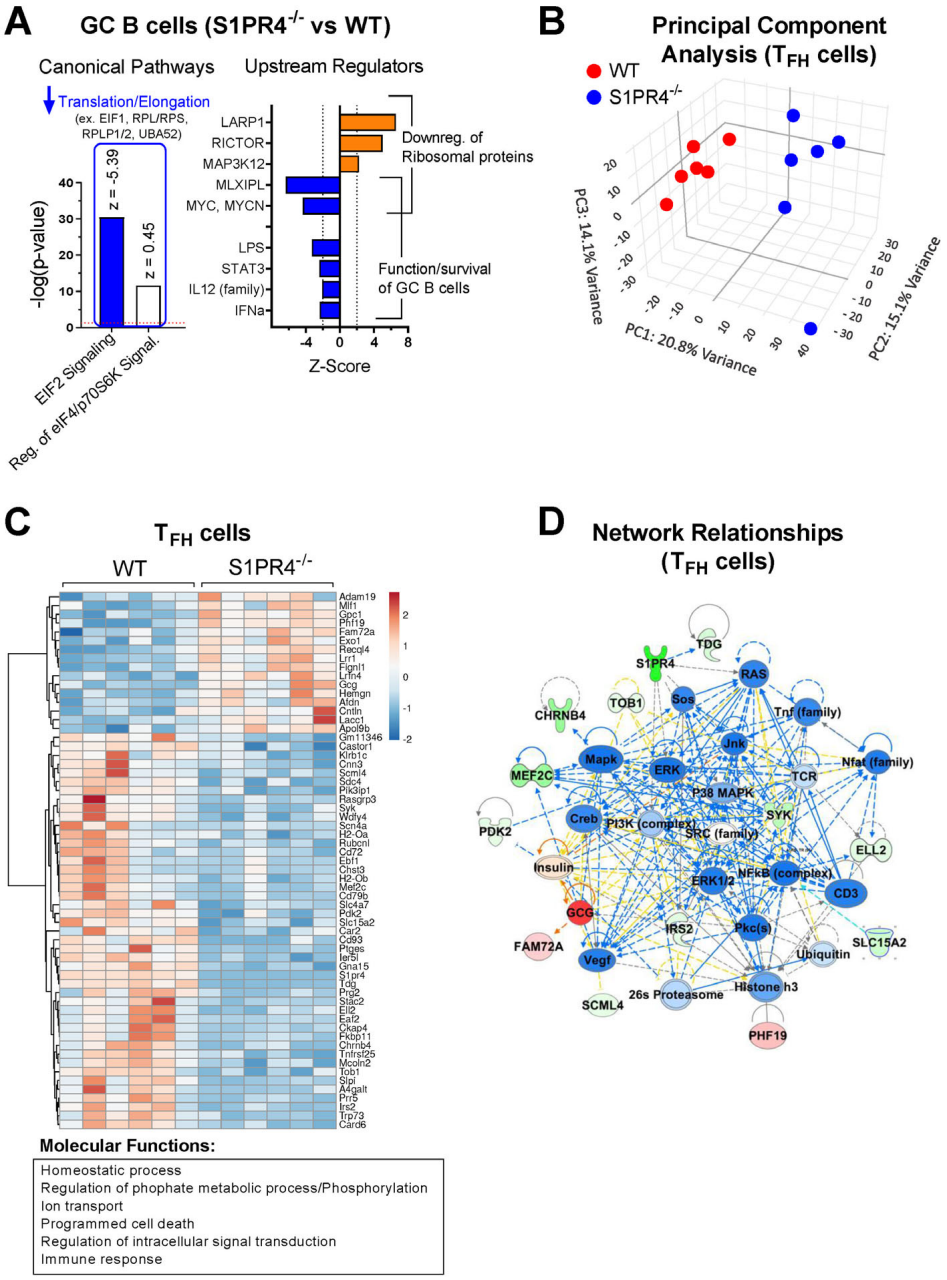
Transcriptome profiling of sorted GC B cells and T_FH_ cells after immunization indicates reduced activation in S1PR4^-/-^ mice. **(A)** RNA-Seq analysis of GC B cells (S1PR4^-/-^ vs WT) isolated nine days after immunization with IFA/LPS/OVA. Analysis was performed using QIAGEN IPA. The top Canonical Pathways with highest p-values are shown on the left and the predicted Z-scores indicated on top of the bars. Blue bar indicates predicted inhibition (negative Z-score), and white bar indicates no prediction of activation/inhibition. Genes affected in the pathways are noted above the bar graph. On the right panel, Z-Scores of Upstream Regulators predicted to be activated (orange bars) or inhibited (blue bars) in S1PR4^-/-^ compared to WT GC B cells with downstream effects indicated on right. **(B)** Principal Component Analysis (PCA) illustrating transcriptional differences between T_FH_ cells isolated from S1PR4^-/-^ and WT dLN six days post-immunization. **(C)** Heatmap of differentially expressed genes (FDR< 0.05 and fold change > 1.5) in T_FH_ cells (S1PR4^-/-^ vs WT) from a subset of genes that are differentially expressed in T_FH_ cells compared to non-T _FH_ CD4^+^ T cells in WT mice (FDR< 0.05 and fold change > 1.5). The top molecular functions related to these genes are indicated on the bottom and were obtained in an enrichment analysis (ShinyGO, http://bioinformatics.sdstate.edu/go/). **(D)** Top Network relationships and predicted functional effects determined by IPA using the genes shown in **(C)** Upregulated genes in the dataset (S1PR4^-/-^ vs WT) are in red; downregulated in green. Predicted inhibited state of molecules are in blue and activated in orange. The degree of changes in expression or activation state is graded by the intensity of the colors. Lines indicate predicted relationships between nodes (blue: leads to inhibition; orange: leads to activation; yellow indicates inconsistent with state of downstream molecule).

To gain further insight into pathways and processes that may be altered in these cell populations, we analyzed the transcriptional landscape of sorted GC B (Day 9) and T_FH_ cells (Day 6) in WT and S1PR4^-/-^ mice at the peak of their respective responses by next-gen RNA-seq. Ingenuity Pathway Analysis (IPA; *p*-value <0.05) of GC B cells identified *EIF2 Signaling* and *Regulation of EIF4/p70S6K Signaling* as the most significantly affected canonical pathways. *EIF2 Signaling* pathway was predicted to be inhibited in S1PR4-deficient GC B cells compared to WT (Z-score = -5.385) (**Figure 2A**, left). Together, these pathways included a group of genes that play a role in the regulation of translation and elongation, such as eukaryotic translation initiation factor 1 (Eif1) and other various ribosomal proteins (**Figure 2A**, left), that were downregulated in S1P4^-/-^ GC B cells, suggesting an impairment in protein synthesis, which may be needed for proper B cell responses to mitogenic signals, including maturation, proliferation, and Ig secretion *in vivo*.

The top upstream regulators predicted by IPA were the RNA binding protein LARP1, RICTOR, MAP3K12, MLXIPL and MYC, with activation scores indicated in **Figure 2A** (right), and whose respective positive or negative activation is consistent with the reduced expression of the translation/elongation genes indicated in **Figure 2A**. In addition to MYC, which is also critical for the formation of GC B cells (46), IPA predicted other upstream regulators with significant negative (inhibited) Z-scores such as LPS, STAT3, IL-12, and IFNα (**Figure 2A**, right), which overall are important for the functionality or activation status of B cells. Of note, comparison of the transcriptome of non-GC B cells indicated that function annotations related to “differentiation of B lymphocytes”, “cell survival and proliferation”, “quantity of cells” and “DNA transcription” were inhibited in S1PR4^-/-^ compared to WT cells (**Supplementary Figure 4A**, left), and that canonical pathways of cell death were activated along with inhibited PI3K/AKT signaling activity pathways (**Supplementary Figure 4A**, right).

Principal component analysis suggested a distinct overall transcriptome in sorted S1PR4^-/-^ T_FH_ cells (**Figure 2B**) where differences between S1PR4^-/-^ and WT T_FH_ cells were not due to altered expression of typical T_FH_ markers such as BCL6, CXCR5, CXCR4, IRF4, MAF, PD1, TCF7, ASCL2, and LEF1 (data accessible through GSE266438). However, among genes differentially expressed in T_FH_ cells, 61 genes exhibited distinct patterns of expression in S1PR4^-/-^ T_FH_ cells (fold change>1.5 and FDR<0.05). In general, these genes were associated with the Gene Ontology (GO) molecular functions of *Immune responses*, *Phosphorylation/Signaling*, *Cellular homeostasis*, and *Cell death* (**Figure 2C**), and their network relationships highlight an inhibition of pathways related to T cell activation (**Figure 2D**). Along these lines, the IPA-predicted inhibition of various upstream regulators, including transcription factors STAT3, STAT5, NF-κb, and cytokines IL-1β, IL-6, TNF, IFNγ, suggests that S1PR4^-/-^ T_FH_ cells may be compromised in their ability to activate, proliferate, and maintain homeostasis (**Supplementary Figure 4B**, center and right). Defective homeostasis-related functions in S1PR4^-/-^ T_FH_ cells were also highlighted by IPA (**Supplementary Figure 4B**, left), suggesting contributing factors to the subsequent reduced GC formation.

The transcriptional landscape of S1PR4^-/-^ GC effector cells was indicative of a stressed environment with impacted function. To investigate whether this weakened state was likely due to cell-intrinsic or -extrinsic factors, we cultured naïve lymphocytes under a variety of conditions. When provided adequate stimulation *in vitro*, S1PR4^-/-^ B cells and CD4+ T cells were functionally indistinct from WT (**Supplementary Figures 5A-D**), demonstrating that S1PR4^-/-^ lymphocytes can mount robust and appropriate responses to immune stimuli. Taken together with the transcriptional and histological analyses, the data suggest that the impaired activation of S1PR4^-/-^ lymphocytes within the GC is likely the result of failed upstream immune events or microenvironmental cues.

### S1PR4-deficiency causes defective early neutrophil mobilization with implications for naïve lymphocyte recruitment and dLN expansion

**Figure 3:**
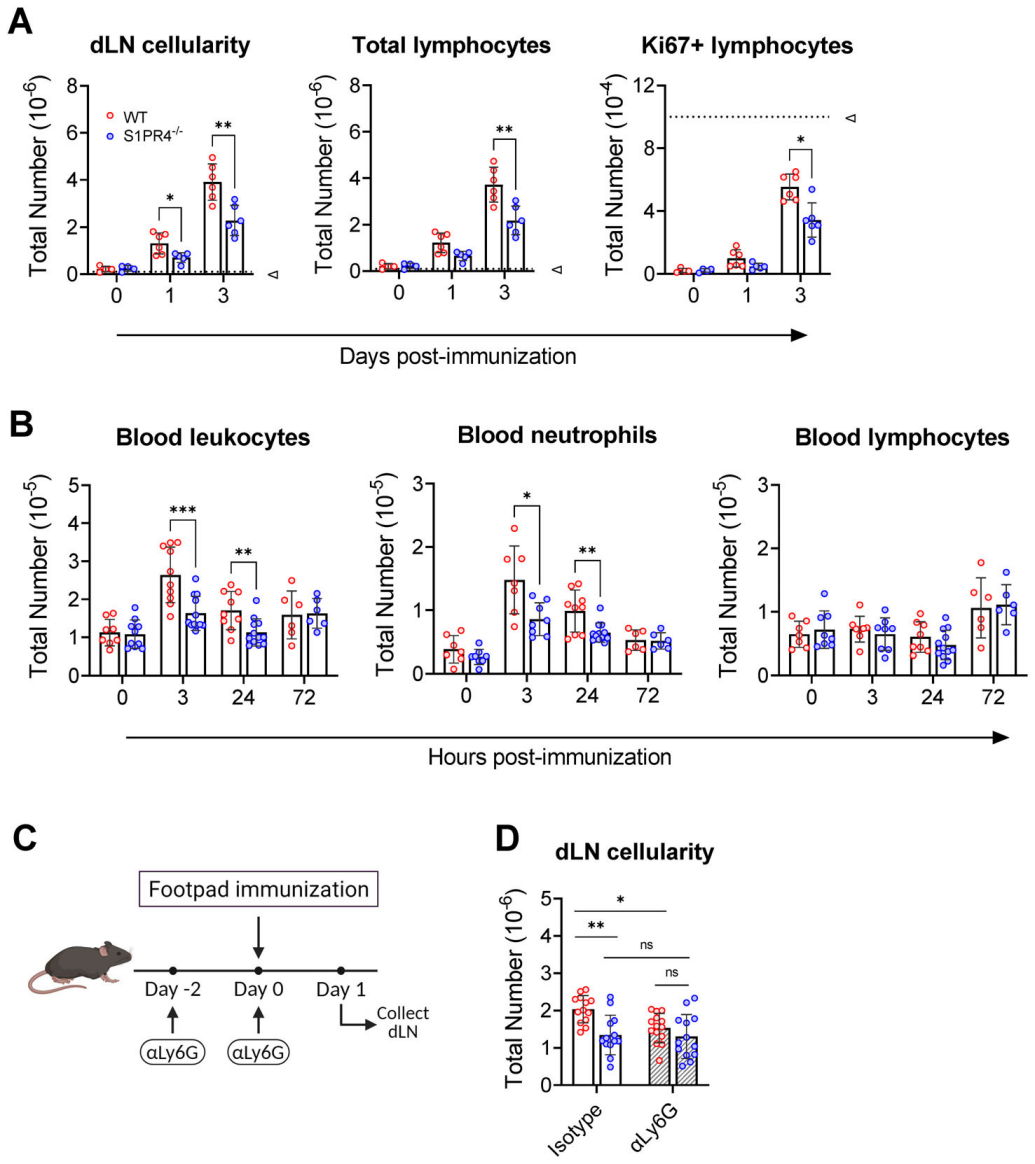
S1PR4 is required for early neutrophil mobilization and subsequent dLN hypertrophy. **(A)** dLN from naïve or immunized mice were evaluated for total cellularity (left), total lymphocytes (middle), and total antigen-respondent (Ki67^+^) lymphocytes (right). Indicated dotted line in all panels represents 10^5^ cells to highlight the change in y-axis scale. **(B)** Total number of circulating leukocytes (left), neutrophils (middle), and lymphocytes (right) quantified by flow cytometry. Data are reported as absolute numbers per 100 µL of whole blood. **(C, D)** Neutrophils mediate early hypercellularity in dLN after immunization. To deplete neutrophils, WT and S1PR4^-/-^ mice were injected with 200 µg anti-Ly6G or isotype control antibody two days prior and at the time of immunization to deplete neutrophils **(C)**. dLN were collected 24 hrs later and evaluated for total cellularity **(D)**. Red and blue circles indicate WT and S1PR4^-/-^, respectively, with a textured bar indicating the neutralizing antibody treatment group. Data represent Mean ± SD of a representative or combined experiment(s) including 4-6 mice and repeated at least three times with similar results; ns, not statistically significant; *p<0.05; **p<0.01; *** p<0.001 using unpaired t-tests.

Lymphocyte infiltration from circulation drives the initial dLN hypertrophy and is thought to prime for an adaptive immune response by increasing the chance of encounter between the exceedingly rare cognate lymphocyte and antigen (Ag) (33). We hypothesized that the GC phenotypes seen at later time points in the response could be, in part, a consequence of impaired early lymphocyte recruitment. In support, we showed that the reduced dLN expansion in S1PR4^-/-^ mice was evident as soon as one to three days post-immunization, with naïve lymphocytes accounting for most of the increase in cellularity (**Figure 3A**, left and center). In agreement, WT mice pre-treated with the S1PR4 antagonist, CYM50358, also resulted in diminished cellular accumulation in the dLN at 24 hrs (**Supplementary Figure 6A**). Furthermore, we found fewer proliferating lymphocytes, as indicated by Ki67 staining, at these early time-points (**Figure 3A**, right), consistent with the hypothesis that S1PR4^-/-^ dLNs were seeded with fewer Ag-respondent cells capable of initiating the downstream GC response. Of note, and as others have reported (33, 47, 48), the extremely low number of Ki67-positive cells indicates that the expanding dLN cellularity at these early time-points is mostly due to the influx of circulating lymphocytes and not the consequence of excessive proliferation. The diminished dLN cellularity in S1PR4-deficient mice, or after antagonism of S1PR4, suggests a role for this receptor in the initial seeding of the dLN to initiate Ag-specific repertoire-screening by circulating lymphocytes.

Although the loss of S1PR4 did not affect circulating leukocyte numbers or frequencies in naïve mice (**Supplementary Table 4**), we wanted to rule out any population abnormalities during immune activation that could explain the diminished recruitment into the dLN from the circulation. Blood analysis at various early time-points revealed a transient increase in total circulating leukocytes by 3 hrs in WT (**Figure 3B**, left), while the change was less noticeable in S1PR4^-/-^ mice. Further investigation revealed that the fluctuation in WT was largely due to an increase in circulating neutrophils, and that this blood neutrophil spike was defective in S1PR4^-/-^ mice (**Figure 3B**, center). This observation, which was confirmed using the S1PR4 antagonist, CYM50358 (**Supplementary Figure 6B**), is in agreement with a described involvement of S1PR4 in neutrophil mobilization (32) and the strong expression of S1PR4 in this cell population (16, 18, 49, 50). In contrast, the levels of circulating lymphocytes remained comparable across these time-points (**Figure 3B**, right).

To determine if the loss of neutrophil mobilization affected subsequent inflammation-induced dLN hypertrophy, we administered a depleting dose of αLy6G antibody two days before, and again just prior to, footpad immunization (**Figure 3C**), effectively eliminating circulating neutrophils by >99% (**Supplementary Figure 7A, B**). Neutrophil depletion resulted in a significant reduction in the number of cells that infiltrated into WT dLN 24 hrs post-immunization, suggesting that early recruitment of naïve lymphocytes into the dLN is neutrophil-dependent. Treatment of S1PR4^-/-^ mice with αLy6G, however, did not alter dLN expansion and thus erased the differences between WT and S1PR4^-/-^ dLN hypertrophy (**Figure 3D**). Together, these results indicate that S1PR4^-/-^ mice harbor an innate defect in early neutrophil mobilization that affects priming of the downstream dLN response.

### Loss of S1PR4 impairs neutrophil infiltration at the immunization site, accompanied by reduced vascular changes

**Figure 4:**
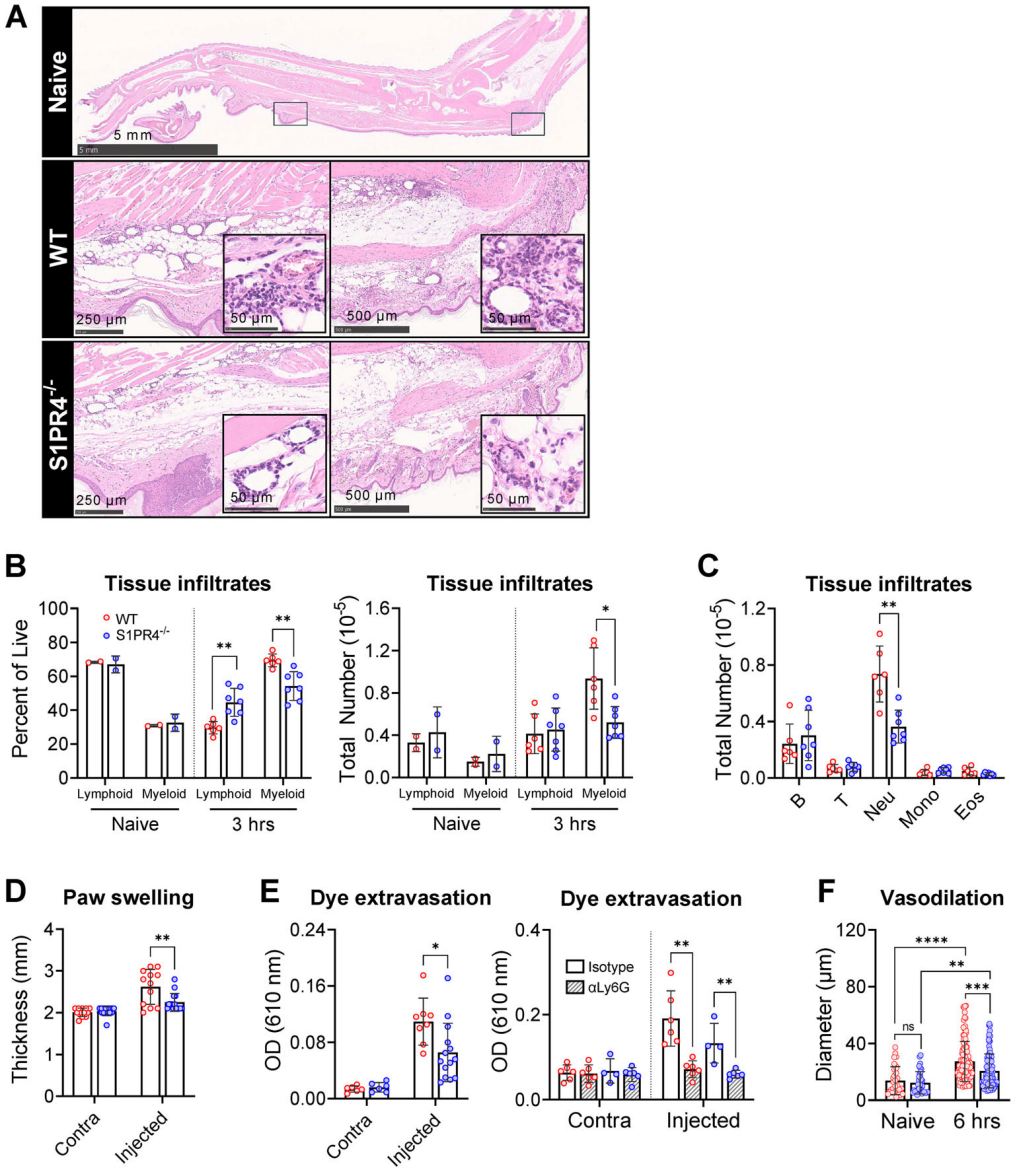
Impaired neutrophil tissue infiltration in S1PR4^-/-^ underlies reduced inflammation and local vascular changes after immunization. **(A)** Hind paws were collected 6 hrs post-immunization then stained with H&E to visualize infiltration by immune cells. The top image is an image of a naïve hind paw indicating with square annotations the mid-planter and heel regions, respectively. Below are representative fields of mid-planter (left) or heel (right) of WT and S1PR4^-/-^ mice, as indicated, at 5X and 80X (insets) magnification, showing early infiltrates. Scale bars indicate 50, 250, 500 µm or 5 mm. **(B, C)** Evaluation of inflammatory infiltrates collected 3 hrs post-immunization via subcutaneous paw flush and analyzed by flow cytometry. **(B)** Percentage (left) and total numbers (right) of lymphoid and myeloid populations from naïve and immunized WT and S1PR4^-/-^ mice, as indicated. **(C)** Differential analysis of the lymphoid and myeloid populations. **(D)** Caliper measurements of experimental and contralateral paw thickness from WT and S1PR4^-/-^ mice 3 hrs post-immunization. **(E)** Vascular permeability at the immunization site was determined via Miles assay. Mice were injected with Evan’s Blue dye 3 hrs post-immunization and tissue collected at 6 hrs. The dye was extracted and OD measurements were performed at 610 nm. On the right panel, mice were treated with neutrophil-depletion antibodies (striped bars) following the protocol shown in **Figure 3C** prior to Evan’s Blue dye injection as in the left panel, then analyzed using a Two-Way ANOVA with multiple comparisons. **(F)** Quantification of vessel diameter from paw sections from **(A)** The data points correspond to individual vessels scored in a total of 4-5 sections, each representing a different mouse. Red and blue circles indicate WT and S1PR4^-/-^, respectively. Data in **(B-F)** represent Mean ± SD of a representative or combined experiment(s) including 4-6 mice and repeated at least three times with similar results; ns, not statistically significant; *p<0.05; **p<0.01; ***p<0.001; ****p<0.0001 using an unpaired t-test unless otherwise stated.

As first responders, neutrophils quickly swarm the site of injury to control infection and initiate the inflammatory cascade. Innate signaling following immunization has been shown to amplify the expansion and remodeling of local vasculature, resulting in altered circulation patterns toward the dLN (33, 45). Because of the diminished neutrophil response in the blood in S1PR4^-/-^ mice and the observed contribution of neutrophils to dLN hypertrophy (**Figures 3B, D**), we sought to characterize the inflammation and vascular changes at the injection site. Compared to naïve mice (**Figure 4A**, top), there was evidence of disrupted tissue and emulsion deposits within immunized paws from our model, as well as pockets of infiltrating immune cells. We observed that the extent of accumulating infiltrates in S1PR4^-/-^ mice was noticeably reduced compared to WT (**Figure 4A**, middle and bottom). Because the infiltrates histologically resembled neutrophils, we performed a subcutaneous paw flush to collect and analyze the post-immunization population changes. While the observed distribution of cells in naïve paws was roughly 70% lymphoid and 30% myeloid (**Figure 4B**, left), there was a drastic inversion in WT mice by 3 hrs due to a rapid accumulation of myeloid cells (**Figure 4B**, right). This shift was largely attenuated in S1PR4^-/-^ mice. Differential analysis at 3 hrs demonstrated a significant reduction in the number of infiltrating neutrophils into S1PR4^-/-^ immunized paws (**Figure 4C**). Similar to other studies (16, 51, 52), a relatively low number of neutrophils migrated to the dLN throughout the model. However, in contrast to the footpad, the total number and frequency of neutrophils in the dLN were not different between WT and S1PR4^-/-^ mice (**Supplementary Figure 7C**).

In parallel to the diminished mobilization and recruitment of neutrophils into the injection site, paw thickness (**Figure 4D**) and dye extravasation into the foot following systemic administration of Evan’s Blue dye (**Figure 4E**, left) were markedly reduced in S1PR4^-/-^ mice, indicating reduced swelling and edema in these mice. Additionally, we found a significant reduction in the extent of vasodilation in S1PR4^-/-^ inflamed paws by measuring vessel diameters in histological sections (**Figure 4F**). Given the known effects of neutrophil activity within the tissue microenvironment, the reduced vessel dilation and permeability in S1PR4^-/-^ mice appears to be a consequence of impaired neutrophil recruitment into the site of injury soon after challenge. Indeed, prior treatment with αLy6G antibodies not only reduced lymphocyte recruitment to the dLN (**Figure 3D**), but also completely prevented inflammation-induced dye leakage at the injection site following challenge (**Figure 4E**, right panel), consistent with a regulatory role for neutrophils in local vascular responses. As changes in vasculature are vital in the progression of inflammation, this may clarify the mechanism behind the neutrophil-mediated attenuation of lymphocyte recruitment into S1PR4^-/-^ dLN.

### Defective dLN hypertrophy is not lymphocyte-intrinsic and culminates with improper late accumulation of dendritic cells and maturation of dLN vasculature

**Figure 5:**
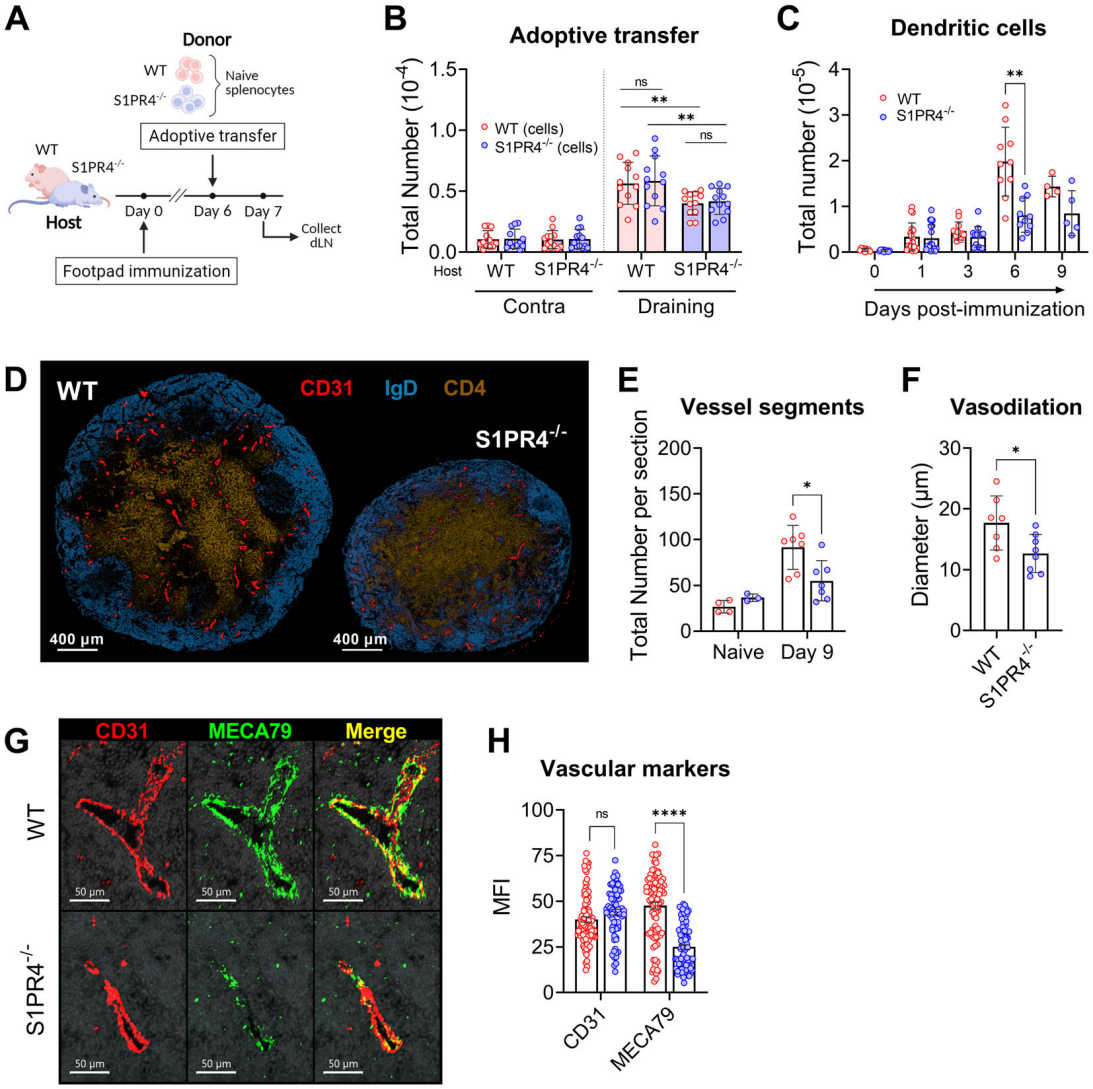
S1PR4-deficiency impairs recruitment of lymphocytes in a lymphocyte-extrinsic manner, late accumulation of dendritic cells, and vascular network development within the dLN. **(A, B)** Fluorescently-labeled WT and S1PR4^-/-^ splenocytes (mixed 1:1) were adoptively transferred into both WT and S1PR4^-/-^ hosts six days post-immunization. 24 hrs later, the number of transferred cells was quantified within the contralateral or draining LN. Pink and blue bar colors indicate WT or S1PR4^-/-^ host, respectively. Donor WT splenocytes are indicated by red circles and S1PR4^-/-^ by blue circles. Data was analyzed using a Two-Way ANOVA with multiple comparisons. **(C)** Quantification of dLN DCs at various time-points post-immunization. **(D)** Representative images of Day 9 dLN with staining for vascular endothelium (CD31, red), B cells (IgD, blue), and T cells (CD4, tan); scale bar, 400 µm. Quantification of **(E)** total identifiable CD31^+^ vessel segments; and **(F)** lumen diameter of largest 20 vessels as a measurement of vasodilation. **(G)** Representative images showing co-localization of vascular endothelium (CD31, red) and HEV regions (MECA79, green) in Day 9 dLN. **(H)** Quantification of the Mean Fluorescence Intensity (MFI) of the CD31 and MECA79 staining from a total of 7-8 sections. Red and blue circles indicate WT and S1PR4^-/-^, respectively. Data shown in B-C includes combined results from at least three independent experiments and error bars represent Mean ± SD; ns, not statistically significant; **p<0.01 using multiple comparisons or multiple unpaired t-tests. Values **(E, F)** represents LN from one individual mouse or multiple measurements within WT or S1PR4^-/-^ LN **(H)**. Error bars represent Mean ± SD **(E, F)** or Mean ± SEM **(H)**; ns, not statistically significant; *p<0.05; ****p<0.0001 using multiple unpaired t-tests unless otherwise stated.

Given that S1PR4 was shown to mediate tissue egress of T cells into lymphatics (22), it was important to evaluate whether S1PR4 also plays an intrinsic role in lymphocyte recruitment from peripheral blood into the dLN during an immune response. To address this question, we adoptively transferred a 1:1 mix of fluorescently labeled WT and S1PR4^-/-^ naïve splenocytes into WT or S1PR4^-/-^ hosts six days post-immunization (**Figure 5A**), a time when the rate of entry of naïve lymphocytes is at its peak (33). A marked increase in the preferential accumulation of transferred lymphocytes into the dLN over the contralateral non-dLN was observed within 24 hrs (**Figure 5B**, left vs right), consistent with previous studies. While this experimental approach confirmed the diminished recruitment of lymphocytes into dLN of S1PR4^-/-^ hosts (**Figure 3A**), we found no difference in the intrinsic homing capability between WT and S1PR4^-/-^ donor cells (**Figure 5B**). Instead, these results suggest there is a lymphocyte-extrinsic factor hindering their recruitment, consistent with our findings involving the contributions of neutrophils.

Thus far, our data has demonstrated a role for S1PR4 in the mobilization of neutrophils to the injection site, local vascular changes, and early seeding of the dLN. The rapid and progressive expansion of an activated dLN, however, is driven by a series of concatenated events involving multiple signals and cell types, and is ultimately supported by an augmented and matured vascular network. For example, signals from infiltrating B lymphocytes into the dLN drive the continued emigration of DCs via the growth of lymphatic vessels (53) and DCs in turn regulate appropriate levels of proliferation and arborization of LN endothelial cells (54–56). Given the attenuated recruitment of B and T lymphocytes into S1PR4^-/-^ dLN (**Supplementary Figure 2A** and **Figure 5B**), we evaluated whether there was a similar reduction in the prolonged accumulation of DCs within the dLN. Early after immunization (one to three days), the absolute number of DCs, similar to that of neutrophils (**Supplementary Figure 7C**), was comparable in WT and S1PR4^-/-^ dLNs. The proper DC migration early on is likely explained by their known tissue-emigration pathway via draining lymphatics in contrast to the recruitment of lymphocytes predominantly through blood venules. Within six days of activity, however, DC accumulation was ultimately hindered in S1PR4^-/-^ mice (**Figure 5C**), consistent with our observation of reduced B lymphocyte entry (**Supplementary Figure 2A**) and the concept of lymphocyte-driven expansion of lymphatics and recruitment of DCs (53).

**Figure 6:**
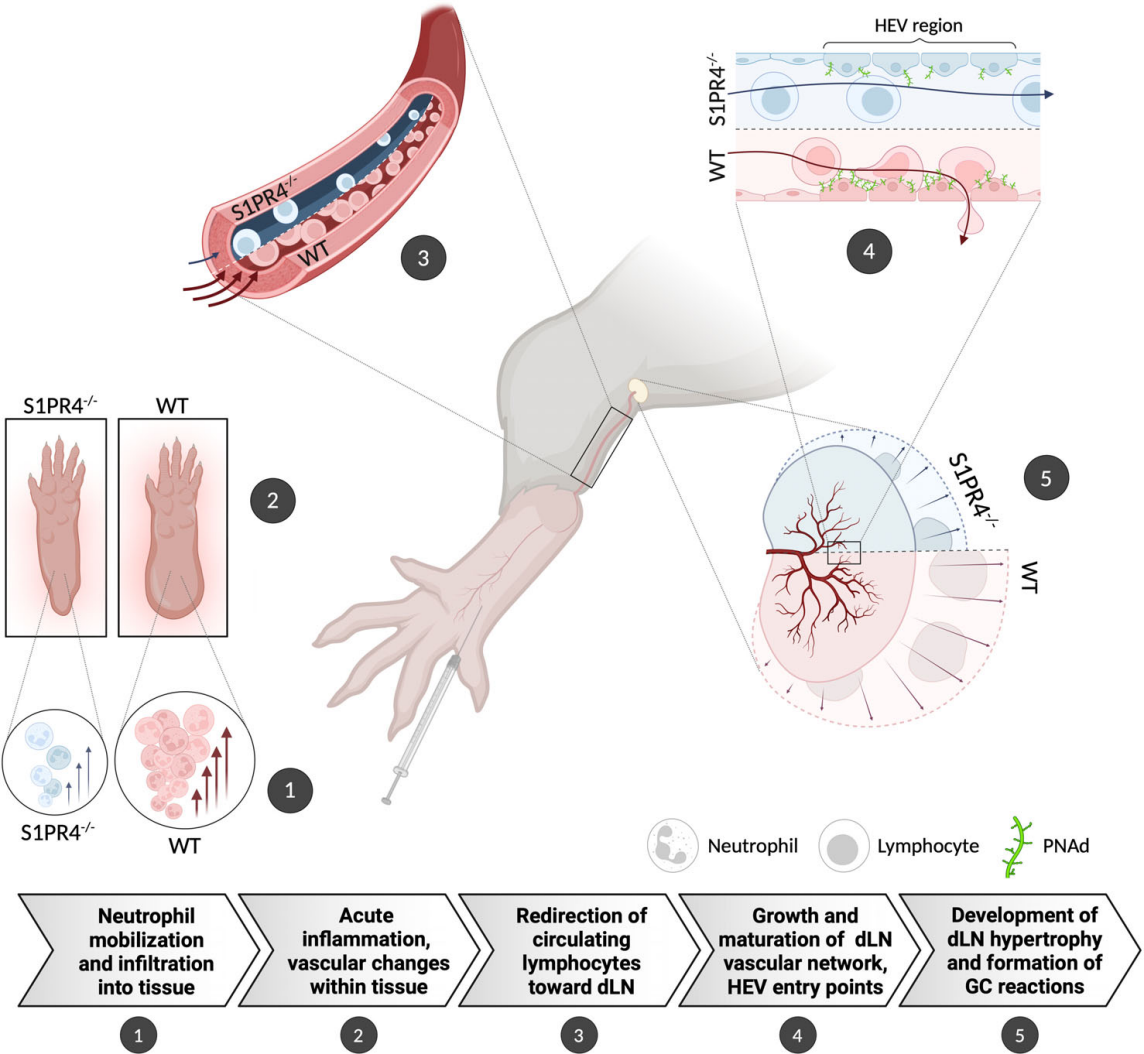
Graphical summary showing the proposed role for S1PR4 in bridging acute inflammation with the downstream formation of a robust GC reaction. (1) Following footpad immunization with IFA/LPS/OVA, neutrophils rapidly mobilize and infiltrate into the site of injection. (2) Increased neutrophils in the tissue initiate an innate response that causes edema and other changes in the local microenvironment. (3) The on-going acute inflammation conveys signals downstream that affect the local vasculature in order to increase circulation toward the dLN. (4) In response, the dLN vascular network begins to expand and mature its HEV portals to accommodate the influx of naïve lymphocytes, thus allowing further entry. (5) The accumulation of circulating lymphocytes results in dLN hypertrophy and allows for the formation of robust GC reactions. In S1PR4^-/-^ mice, the amplification of this immune cascade is impaired, in part, due to failed neutrophil recruitment. Consequently, these mice exhibit reduced immune activity that ultimately results in attenuated GC formation. The graphic depicts the differing WT and S1PR4^-/-^ phenotypes in red and blue, respectively.

Because DCs play a role in regulating the adequate growth of the capillary network, we next evaluated histological sections at the Day 9 peak of the response (**Figure 5D**). In S1PR4^-/-^ dLN we found a significant reduction in the number of identifiable CD31^+^ vessel segments (**Figure 5E** and **Supplementary Figure 8A**), as well a narrower lumen diameter (**Figure 5F**) among the largest vessels, indicative of reduced development and arborization of the vascular network. We also observed reduced staining intensity of MECA79 (**Figures 5G, H**), which is used to identify high endothelial venule (HEV) regions. Because MECA79 recognizes the ligand for L-selectin, peripheral node addressin (PNAd), the reduced availability at S1PR4^-/-^ entry points limits adhesion by rolling lymphocyte, representing a major impediment for dLN infiltration (57). Of note, we found in our RNAscope data that the expression of *S1pr4* is very low in vessel endothelial cells of the activated dLN, compared to the high expression of *S1pr1* (**Supplementary Figure 8B**). In support, analysis using data from single-cell sequencing repositories also indicates that expression of *S1pr4* is minimal in vascular endothelial cells from various tissues (**Supplementary Figure 8C**) and in HEVs under homeostatic or inflamed conditions (**Supplementary Figure 8D**), thus making an intrinsic defect within the vascular compartment in S1PR4^-/-^ mice unlikely. Rather, the sum of our data here suggests that dLN hypertrophy, which ultimately functions to seed GC reactions with Ag-respondent cells, is compromised in mice lacking S1PR4 due to upstream innate deficiencies, particularly early neutrophil mobilization, leading to eventual suppression of lymphocyte recruitment and growth-driven vascular arborization (**Figure 6**).

## Discussion

Unlike other members of the S1PR family, expression of S1PR4 is predominantly confined to the hematopoietic compartment. Despite multiple studies revealing regulatory functions for S1PR4 in a variety of innate and adaptive immune cell types, an understanding of how S1PR4 affects the progression of an overall immune response remains incomplete. Although a recent report found that S1PR4-deficiency in a sepsis model caused reduced splenic GC formation (23), the mechanisms leading to these abnormalities were ambiguous. Here, using a local T_H_1 immunization model, we perform detailed studies on the progression of LN hypertrophy and GC formation in S1PR4^-/-^ mice. We present evidence of a critical role for S1PR4 in bridging neutrophil-driven innate inflammation to the lymphocyte recruitment necessary for dLN expansion and growth-driven maturation necessary to support a robust GC reaction (as summarized in **Figure 6**).

One of the most striking features of the S1PR4^-/-^ phenotype was a marked reduction in dLN size, which occurred in parallel to the formation of fewer, smaller, and less populated GCs than WT mice (**Figure 1**). By carefully monitoring the progression, we found that the S1PR4^-/-^ response did not attain the sharp peak seen in WT between six to nine days post-immunization, even at later times. Instead, the attenuated plateau suggests a diminished magnitude, rather than a delay in the response (**Figure 1A**). We further established that early differences in dLN cellularity were not a consequence of impaired proliferation, as clonal expansion does not appreciably contribute to cellularity until much later in the response (33, 47, 48). Rather, our data is most consistent with a failure of naïve lymphocytes to home from blood to the dLN (**Figure 3A**). Through adoptive transfer experiments, we found this impaired migration to likely be lymphocyte-extrinsic since both WT and S1PR4^-/-^ splenocytes were similarly able to enter dLN in WT hosts (**Figure 5B**). This is consistent with other studies concluding that S1PR4 deficiency has little effect on lymphocyte migration toward S1P *in vitro* or *in vivo (* 20, 21, 23, 27), although others have found that S1PR4 contributes to CD4^+^ T cell entry into afferent lymphatics (22) and the migration of peritoneal B1 cells into peripheral secondary lymphoid organs (20, 58, 59). Critically, the increased flow of naïve lymphocytes toward an activated dLN is a key process to maximize repertoire-screening required for an effective adaptive immune response against an Ag. The defective recruitment seen in S1PR4^-/-^ mice likely resulted in the identification of fewer Ag-respondent B and T cells. With fewer effector cells available to seed cognate interactions, we conclude that S1PR4^-/-^ mice ultimately develop a limited number of GC reactions in the dLN, similar to the reduced splenic follicle sizes in the sepsis model described by Riese (23).

Consistent with this conclusion, our RNA-Seq analysis on sorted dLN effector cells indicated suboptimal activation of GC B cells and T_FH_ cells in S1PR4^-/-^ mice at the Day 9 and Day 6 peak of their respective population responses (**Figure 2** and **Supplementary Figure 4**). For instance, the downregulation of ribosomal proteins and translational programs in S1PR4^-/-^ GC B cells may be linked to negative effects on proliferation and cytokine secretion, as described in other systems (60). Furthermore, the activities of known critical regulators of GC B cell differentiation, such as MYC (46) and STAT3 (61), were predicted to be downregulated compared to WT. In T_FH_ cells from S1PR4^-/-^ mice, the distinct transcriptional profile included a number of genes whose network interactions also suggest a reduced state of activation and cytokine production compared to WT. Despite these differences that developed *in vivo*, we found that naive B cells and CD4^+^ T cells were able to properly mature and activate *in vitro*, as others have reported (27). While this suggests that lymphocyte-intrinsic S1PR4 is not required for differentiation under these particular culture conditions, we cannot rule out a potential role for S1PR4 signaling within the microenvironment of the dLN that is not manifested under our optimal conditions in culture. For instance, a study found that loss of S1PR4 in splenic B cells resulted in reduced chemotaxis toward CXCL13, although it did not affect migration to S1P (23), and thus it is also possible that absence of S1PR4 has unexpected consequences on responses to other signals *in situ*. Of interest, we observed that *S1pr4* mRNA expression, as that of *S1pr1*, was abundant in B and T cells throughout the activated LN but nearly absent in the GC areas (**Supplementary Figure 3**). This is similar to the pattern of expression of other regulatory proteins, including S1PR1, EBI2, KLF2 (12, 62, 63), known to affect positioning, retention, or differentiation fates of T_FH_ or GC B cells. We also observed that S1PR4^-/-^ CD4^+^ T cells were located further from the border of the GC than in WT (**Supplementary Figure 8**). While this finding could be consistent with altered migration patterns or increased retention in these areas, the difference was not prominent and would need confirmation through further investigation. Despite such possibilities, we believe the deficient GC formation and dLN expansion can be generally explained by the diminished lymphocyte recruitment resulting from an early upstream event in the immune response rather than major intrinsic defects in S1PR4^-/-^ lymphocyte homing or differentiation in this model.

An important observation in our study was the link between early neutrophil infiltration and the redirection of naïve lymphocytes to the dLN. Neutrophils are the first cells to invade the affected tissue where they create a highly pro-inflammatory microenvironment through the release of cytokines, lytic enzymes, and reactive oxygen species that lead to immune cell recruitment and activation (64, 65). This acute inflammation not only helps to neutralize microbes at the site of injury but may also prime the dLN for a robust adaptive immune response. In our model, we observed an early spike of neutrophils in circulation and the footpad, along with vascular changes in the tissue, all of which were severely diminished in the absence of S1PR4 or after S1PR4 antagonism (**Figures 3B**, **4**, and **Supplementary Figure 6**), demonstrating that S1PR4 helps regulate neutrophil mobilization into tissue after immunization. Furthermore, when neutrophils were depleted, vascular leakage (**Figure 4E**) and the number of cells accumulating in the dLN at 24 hrs post-immunization (**Figure 3D**) were both effectively reduced, indicating a role for neutrophils in promoting local changes in tissue vasculature and in the early recruitment of circulating lymphocytes into the dLN. Based on studies demonstrating that during inflammation, innate signal-induced vascular remodeling is critical for naïve lymphocyte recruitment to the dLN (33), and that neutrophils can directly or indirectly affect the vascular compartment (66–69), we postulate that the mechanism by which neutrophils promote lymphocyte seeding relates to vasculature alterations initiated within the injection site. Consistent with this, we found that the small number of neutrophils accumulating within the dLN was not different between WT and S1PR4^-/-^ mice (**Supplementary Figure 7C**), suggesting their role lay upstream of the dLN. Although this potential causal relationship needs demonstration, the concept is also supported by other studies showing that neutrophils mediate tumor angiogenesis (66, 67), and lymphangiogenesis in the footpad and LN in immunized mice by promoting the bioavailability of VEGF (68), a potent angiogenic factor also critical for the development of the LN vascular network (53, 54). Regardless of the mechanism, our data suggests the dampened early neutrophil-mediated inflammation in our model minimized dLN growth of S1PR4^-/-^ mice, likely impacting the repertoire-screening required to seed GC reactions with Ag-respondent lymphocytes recruited out of circulation (**Figure 3**).

Inflammation-induced expansion of the dLN, however, is a well-orchestrated process involving many signals and cell populations. In order to accommodate the enhanced cellular influx, the dLN must increase HEV entry points and also grow its capillary network to maintain an adequate supply of oxygen, nutrients, and waste removal (56). By Day 9 when GCs are fully formed, the extent of vascular development was noticeably diminished in S1PR4^-/-^ mice (**Figures 5D–F**), indicating a disadvantageous environment meant to support an active immune response. Furthermore, endothelial expression within HEV regions of the lymphocyte adhesion ligand, PNAd (57, 70), which defines mature HEVs and supports homing of lymphocytes, was also greatly reduced in the dLN of immunized S1PR4^-/-^ mice (**Figures 5G, H**). While our data suggest that neutrophils mediate the selective redirection of lymphocytes early in the response, it is likely that this lower degree of HEV maturity further antagonized their recruitment. Another innate cell type intimately involved in the coordination of dLN expansion is the DC. These infiltrating cells are critical not only for Ag-presentation and the maturation of HEV networks, but also in facilitating continued dLN expansion (54, 70). It has been shown that dLN entry of lymphocytes and DC are intertwined through a positive feed-forward loop whereby the increased influx of one population enhances the recruitment of the other (53). Our findings indicate that while initial migration of DCs to the dLN was not affected by loss of S1PR4, maximal DC accumulation later in the response was compromised (**Figure 5C**). While this could be a consequence of the defective early lymphocyte entry, reciprocally the reduced DC accumulation leading to improper HEV expansion, may compound to the diminished lymphocyte recruitment. The reduction in maximal DC accumulation contrasts with the comparable levels in S1PR4^-/-^ and WT dLN of infiltrating neutrophils (**Supplementary Figure 7C**), where no such recruitment synergy with lymphocytes has been reported. Overall, it is likely that other abnormalities within the innate compartment culminates in the phenotype seen in S1PR4^-/-^ mice, with minimal or no lymphocyte-intrinsic defects, as summarized in **Figure 6**.

Even though this study revealed unique functions of S1PR4 that become evident upon genetic deletion or chemical inhibition, other roles for S1PR4 during inflammation may be masked by functional redundancy with other S1PRs. Additionally, we cannot completely exclude the possibility that changes in the expression of other S1PR family members upon S1PR4 ablation may contribute to phenotype described herein. We find this unlikely, however, as the included RNA-Seq data from lymphocyte populations (available in the GSE266438 repository) and our prior study in mast cells (26), did not reveal any major repercussions in the level of mRNA expression of other S1PR family members.

Altogether, our study shows that in a model of local T_H_1 inflammation, loss of S1PR4 negatively impacts neutrophil mobilization and infiltration into the injection site, resulting in reduced acute inflammation, and impaired lymphocyte-driven dLN hypertrophy. Defective recruitment at early stages likely fails to equip the dLN with Ag-respondent lymphocytes necessary for productive cognate interactions. We believe the data supports the conclusion that the severe impact on GC formation observed in S1PR4^-/-^ mice is a consequence of a series of concatenated events, driven primarily by innate components, that profoundly impacts the magnitude of GC reactions during the development of the downstream adaptive immune response.

## Data Availability

The datasets presented in this study can be found in online repositories. The names of the repository/repositories and accession number(s) can be found below: GSE266438 (GEO).
