# Supplementary material for: Sphingosine-1-Phosphate Receptor 4 links neutrophils and early local inflammation to lymphocyte recruitment into the draining lymph node to facilitate robust germinal center formation
Source: Front Immunol. 2024 Aug 12;15:1427509. doi: 10.3389/fimmu.2024.1427509 (PMC11345157; doi:10.3389/fimmu.2024.1427509)
Supplement: Supplementary file 1 [file DataSheet_1.pdf]

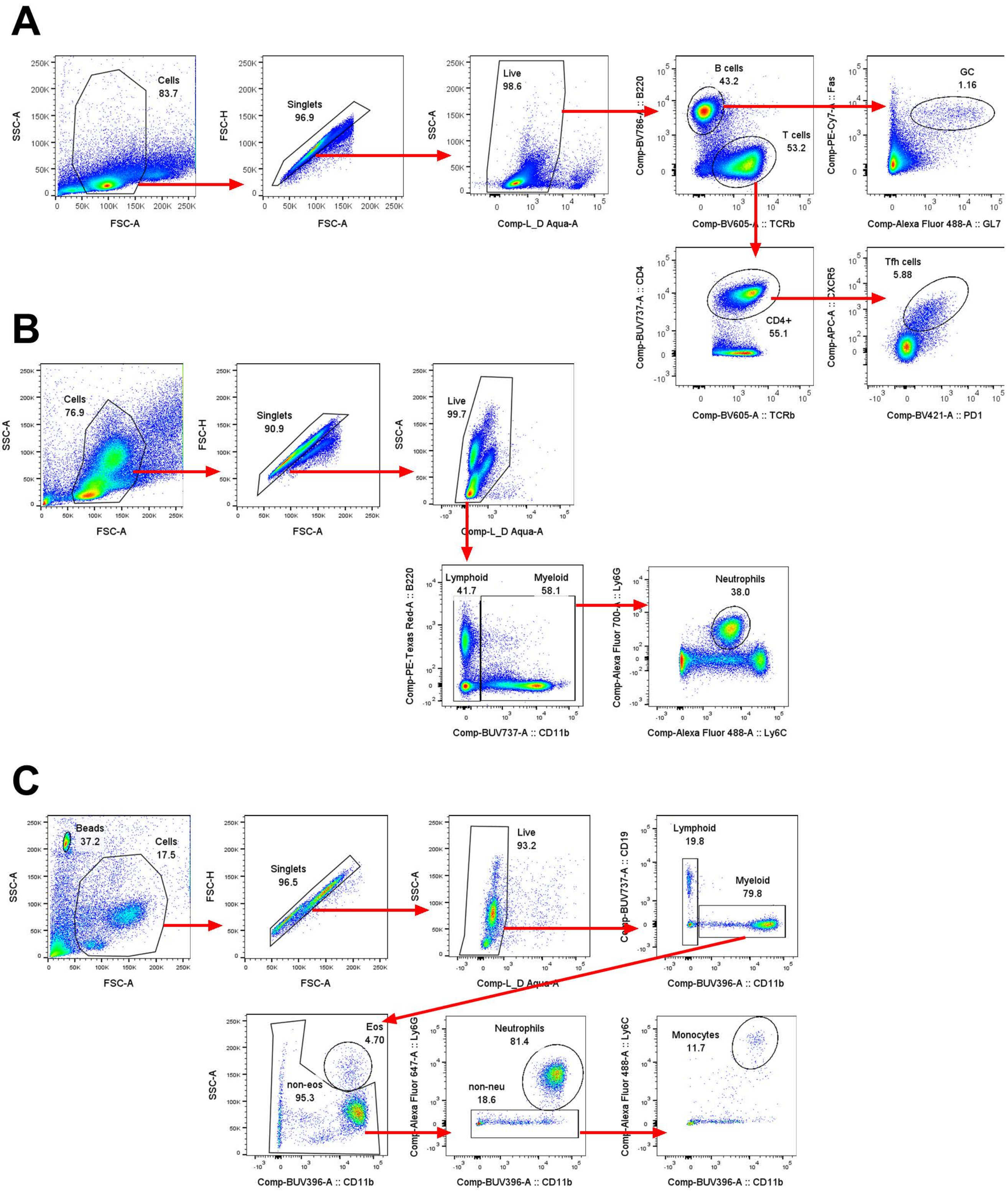

Supplemental Figure 1. Gating strategies for flow cytometric analysis. Examples showing the gating process used during the analysis of **(A)** dLN, **(B)** blood, and **(C)** subcutaneous foot flush samples.

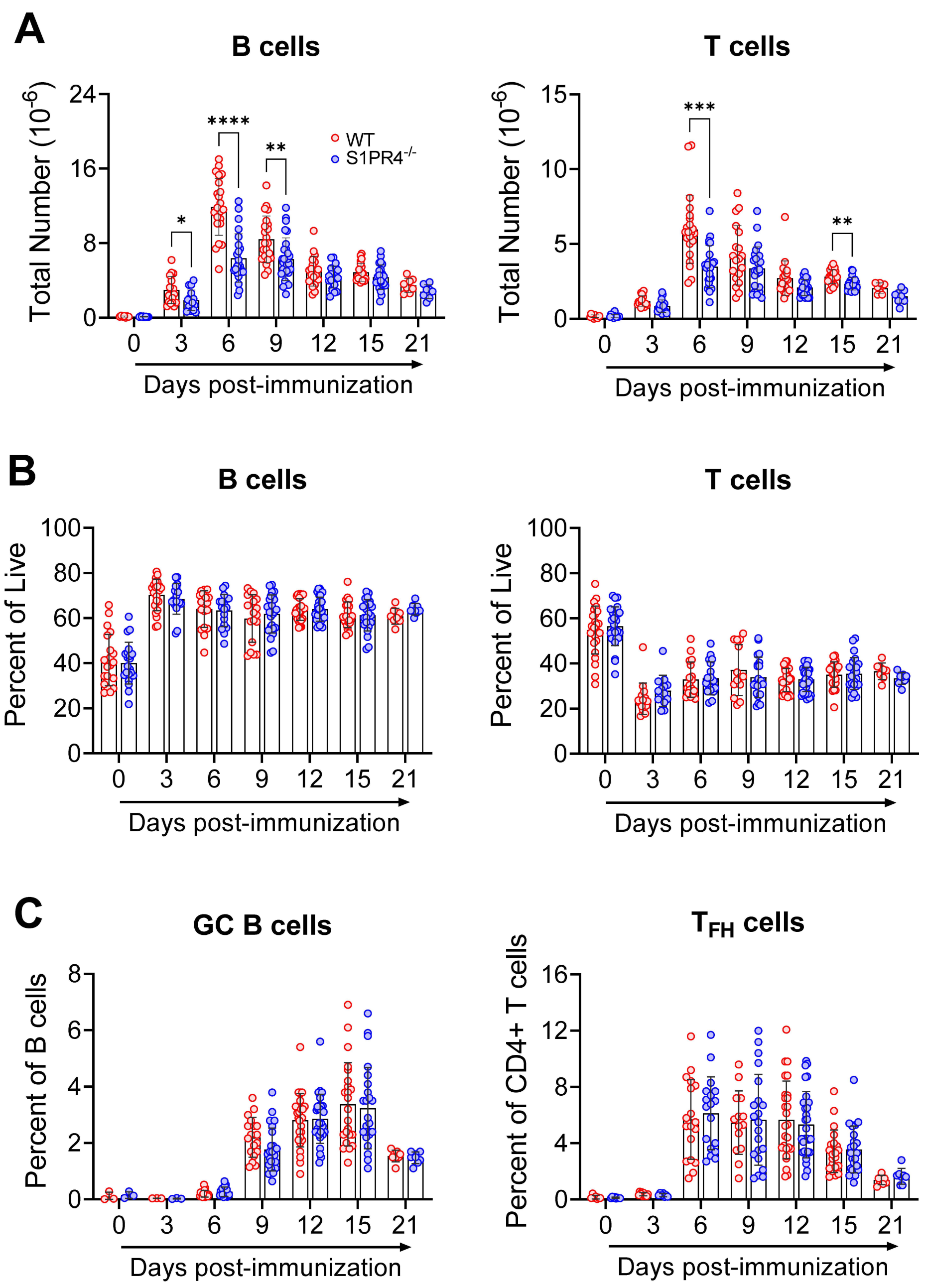

Supplemental Figure 2. Reduced lymphocyte numbers and GC responses in S1PR4$^{-/-}$ mice do not result in changes in the frequency of specific populations. dLN from footpad-immunized WT and S1PR4$^{-/-}$ mice were collected at various time-points and analyzed by flow cytometry for **(A)** total number and **(B)** frequency of B (left) and T (right) cells. **(C)** Frequency of GC B cells among total B cells (left), and frequency of T_{FH} cells among CD4+ T cells (right). Values represent data from combined experiments repeated at least three times with similar results. Bars represent Mean ± SD; *p<0.05; **p<0.01; ****p<0.0001 using multiple unpaired t-tests.

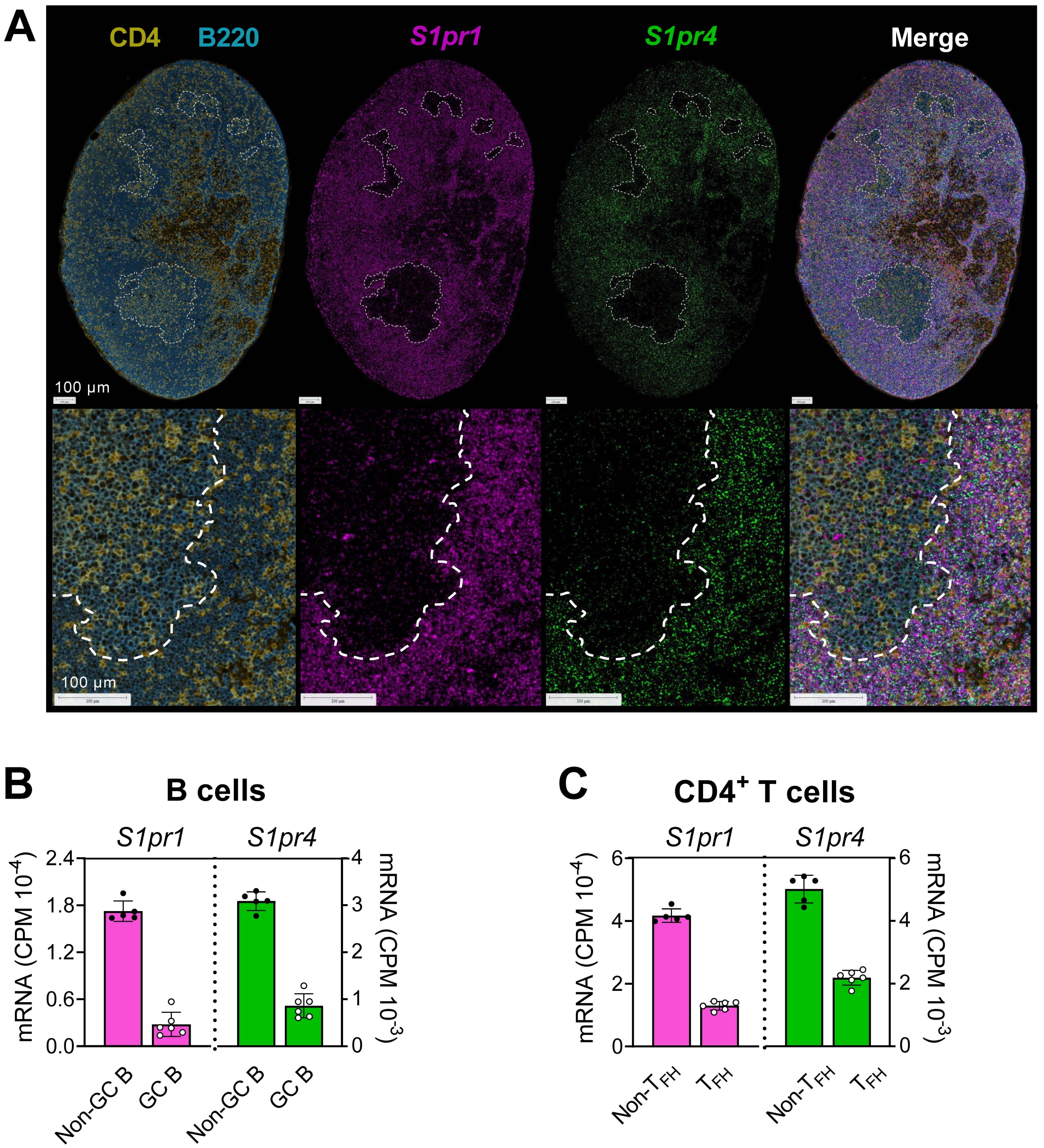

Supplemental Figure 3. ***In situ*** **profiling of** ***S1pr4*** **mRNA expression in dLN.** **(A)** RNAscope *in situ* hybridization illustrating the spatial distribution of mRNA for *S1pr1* (magenta) and *S1pr4* (green) in WT dLNs nine days post-immunization with IFA/OVA/LPS. LN sections were also multiplexed to detect $CD4^+$ T cells (tan) and $B220^+$ B cells (blue) by immunohistochemistry. Dotted white outline delineates GC regions. Scale bar, 100 µm. **(B)** Raw reads of *S1pr1* and *S1pr4* transcripts in the indicated sorted dLN populations after immunization as determined by RNA-seq and expressed as counts per million (CPM) mapped reads. GC B cells and non-GC B cells were isolated on Day 9 after immunization, and T_{FH} and non-T_{FH} CD4+ T cells on Day 6. Data represents Mean ± SD. Differences in transcript levels between GC B cells and non-GC B cells or between T_{FH} and non-T_{FH} CD4+ T cells were all statistically significant with p values <0.0001 and FDR <0.05.

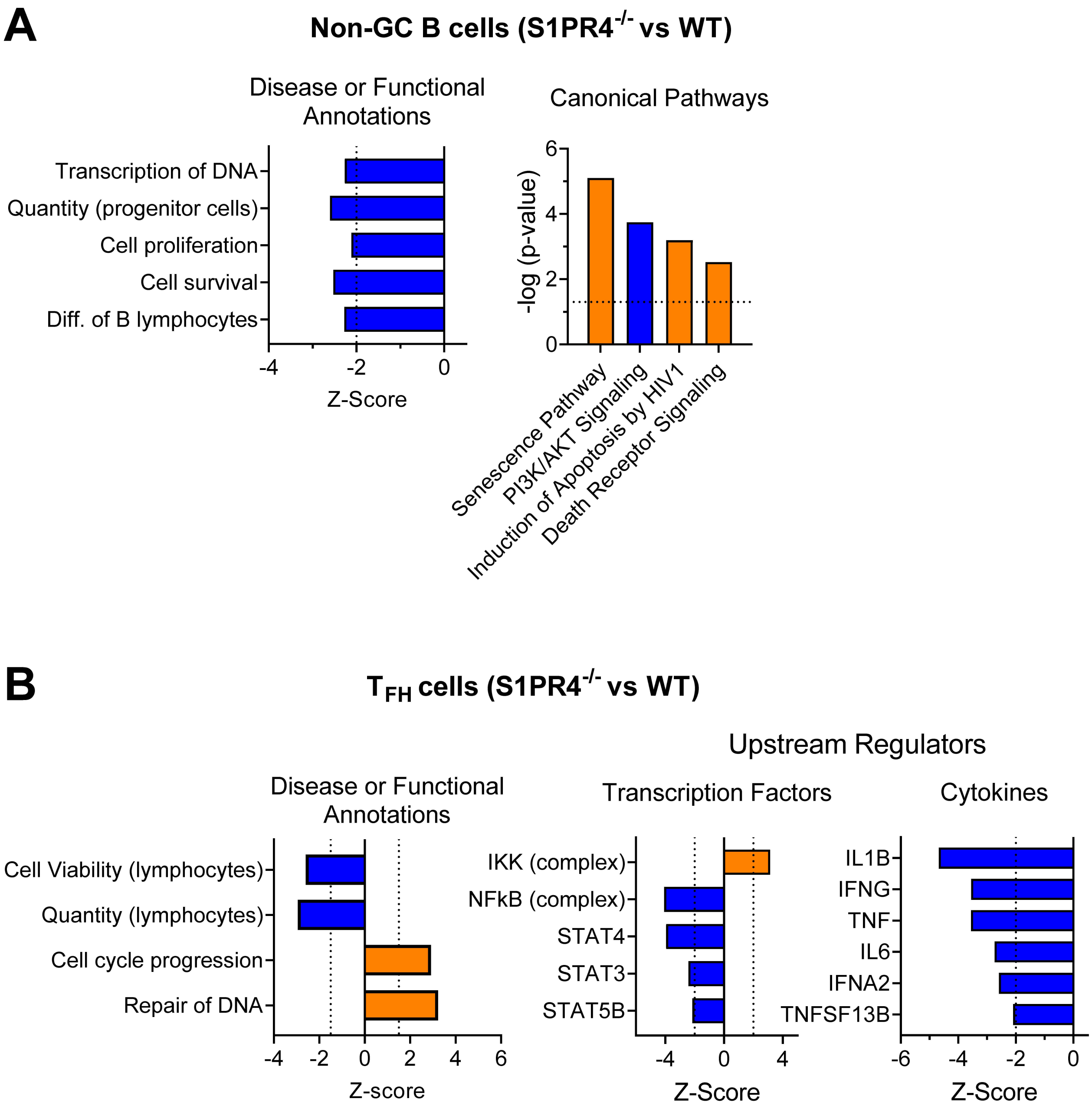

Supplemental Figure 4. Transcriptional analysis of LN populations during an immune response. **(A)** Transcriptional analysis of sorted non-GC B cells from immunized S1PR4$^{-/-}$ mice compared to WT mice (Day 9) using QIAGEN IPA. Shown are Diseases or Functions Annotations (left) and Canonical Pathways (right) predicted to be significantly activated or inhibited as indicated by the Z-scores and colors (activated: orange bars; inhibited: blue bars). **(B)** Transcriptional analysis of sorted T_{FH} cells from immunized S1PR4$^{-/-}$ compared to WT mice (Day 6) using QIAGEN IPA. Shown are Disease or Functional Annotations (left) and predicted Upstream Regulators (center and right) with positive or negative Z-scores, as indicated.

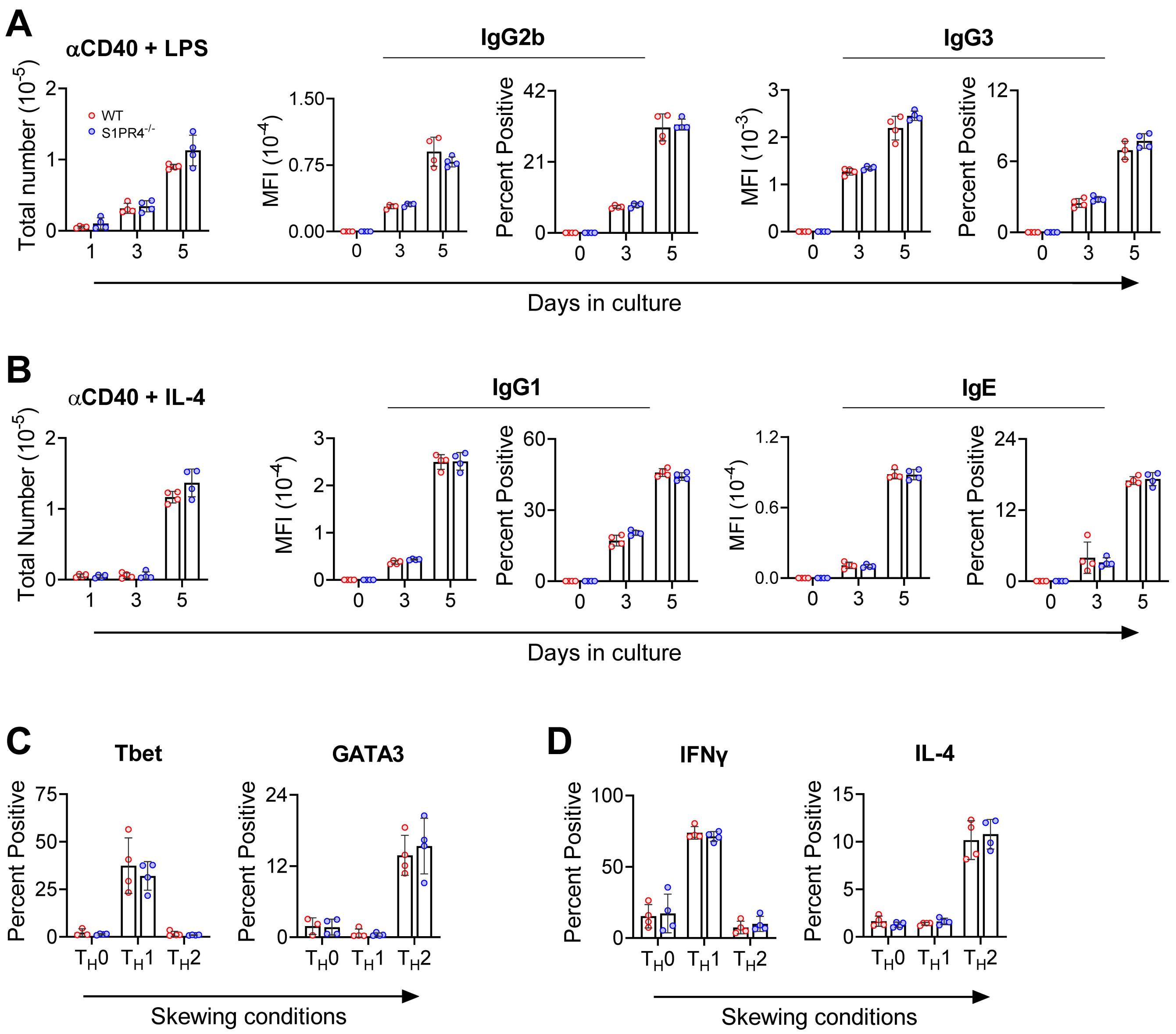

Supplemental Figure 5. S1PR4$^{-/-}$ lymphocytes activate normally *in vitro* under various culture conditions. Naïve splenic B cells were isolated and cultured with **(A)** anti-CD40 and LPS or **(B)** anti-CD40 and IL-4 for five days. Total cell numbers per wells (left panels) and production of the indicated antibodies (middle and right panels) as measured by intracellular flow cytometry were determined 3 and 5 days after culture initiation. **(C-D)** Naïve splenic CD4^{+} T cells were cultured with anti-CD3/anti-CD28 and IL-2 alone (T_H0) or in the presence of T_H1 or T_H2 skewing cocktails, as indicated, for five days. Following activation with PMA+Ionomycin, the expression of **(C)** transcription factors T-bet (T_H1) and GATA3 (T_H2) and **(D)** cytokines IFNγ (T_H1) and IL-4 (T_H2) were measured by trans-nuclear or intracellular flow cytometry, respectively. Data are from a representative experiment repeated three times with similar results. Bars represent Mean ± SD.

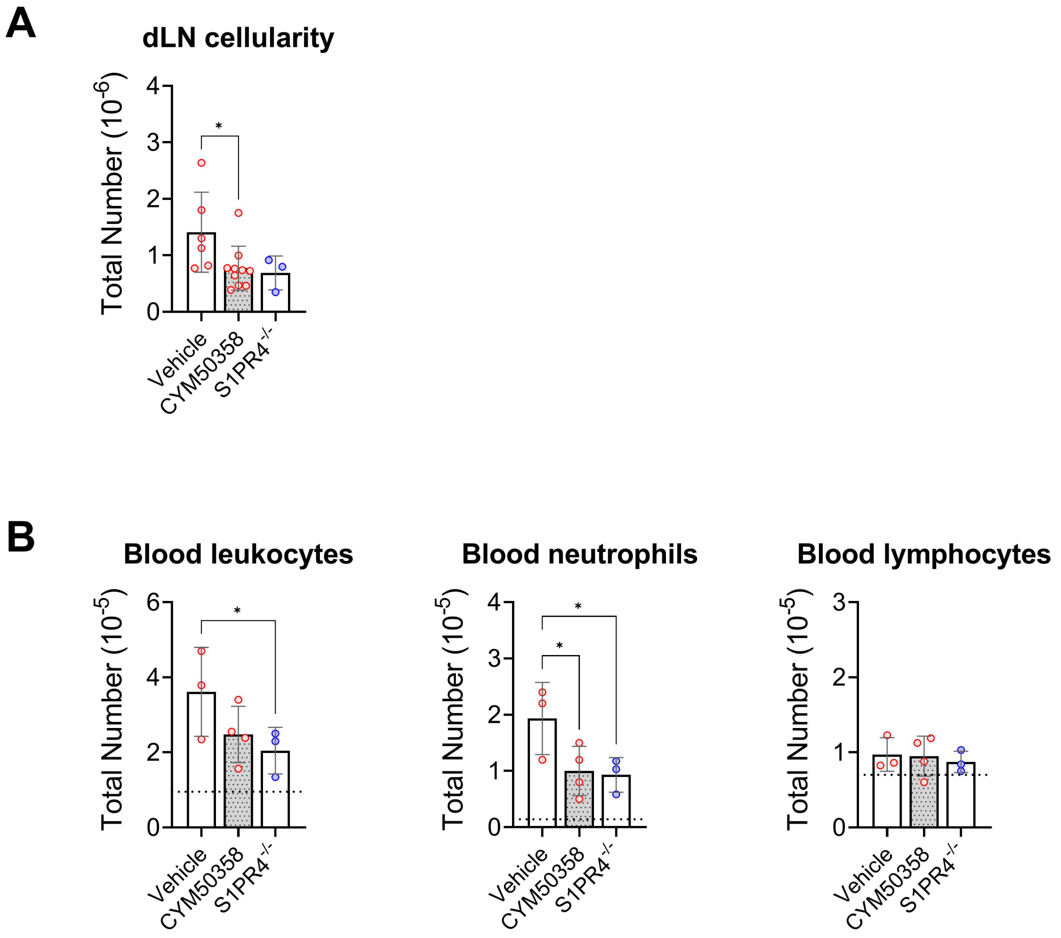

Supplemental Figure 6. Similar to S1PR4$^{-/-}$ mice, inhibition of S1PR4 by the specific antagonist CYM50358 reduces early inflammation in the dLN and injection site. Mice were injected with 10 mg/kg of CYM50358 30 min prior to footpad injection. **(A)** dLN cellularity at 24 hrs. **(B)** Total number of circulating leukocytes (left), neutrophils (middle), and lymphocytes (right) in 100 µL blood at 3 hrs post-immunization. Dotted line represents values in naive mice. Red and blue circles indicate WT and S1PR4$^{-/-}$, respectively, with a shaded bar indicating antagonist-treated group. Data represent Mean ± SD of a representative or combined experiment(s) repeated twice with similar results with 3-4 mice in each group. *$p<0.05$ using unpaired t-tests.

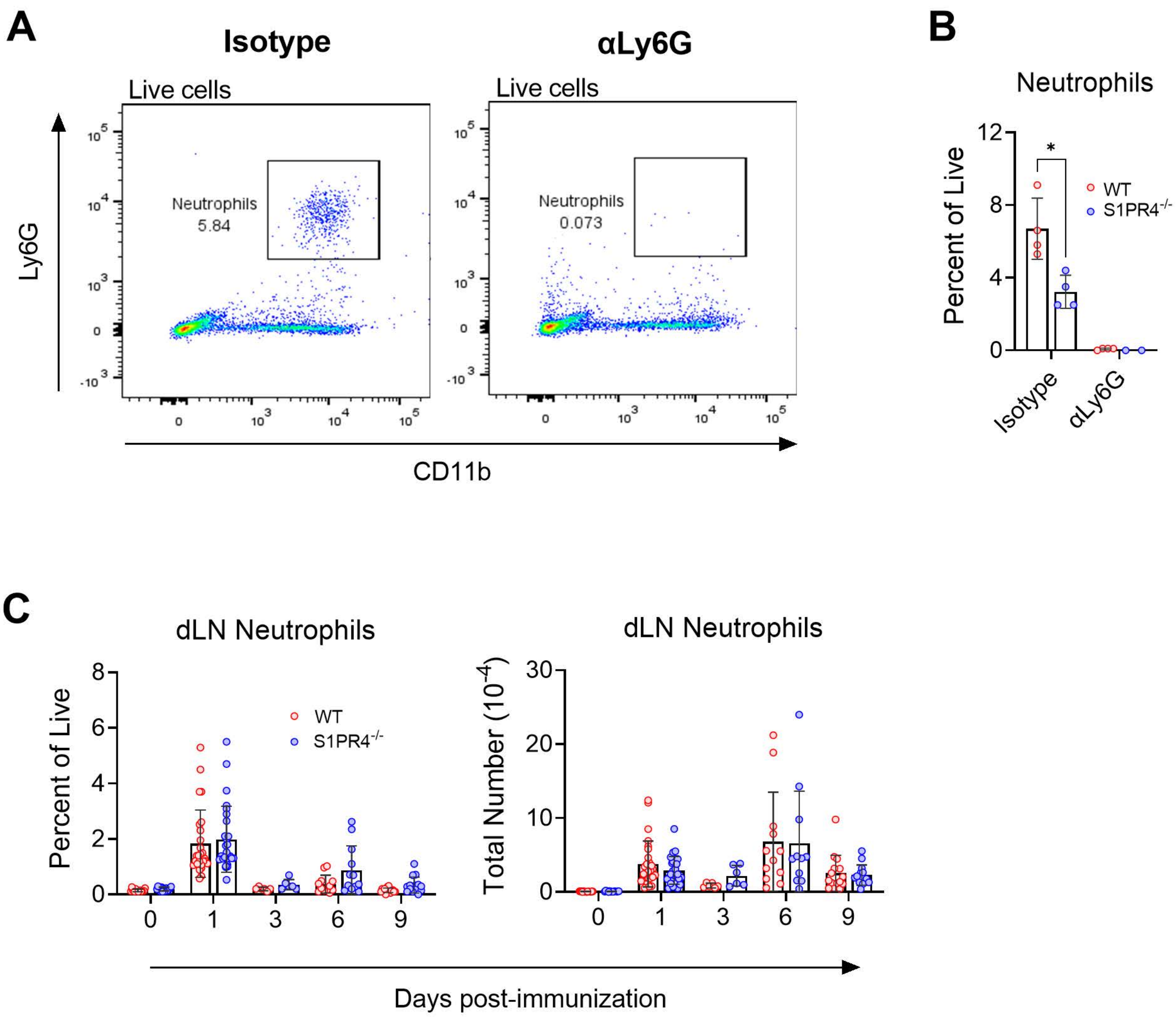

Supplemental Figure 7. Demonstration of neutrophil depletion after treatment with anti-Ly6G and neutrophil quantification within dLN. Mice were injected with 200 µg anti-Ly6G or isotype control antibody on Day -2 and 0 before footpad immunization. 24 hrs post-immunization, blood was collected and analyzed for the presence of neutrophils. **(A)** Representative flow cytometric plots and **(B)** quantification showing successful depletion of circulating neutrophils. **(C)** Time-course data showing the percent (left) and total number (right) of neutrophils present in the dLN at the indicated times post-immunization. Values in C represent data from combined experiments repeated at least three times with similar results. Data represents Mean ± SD.

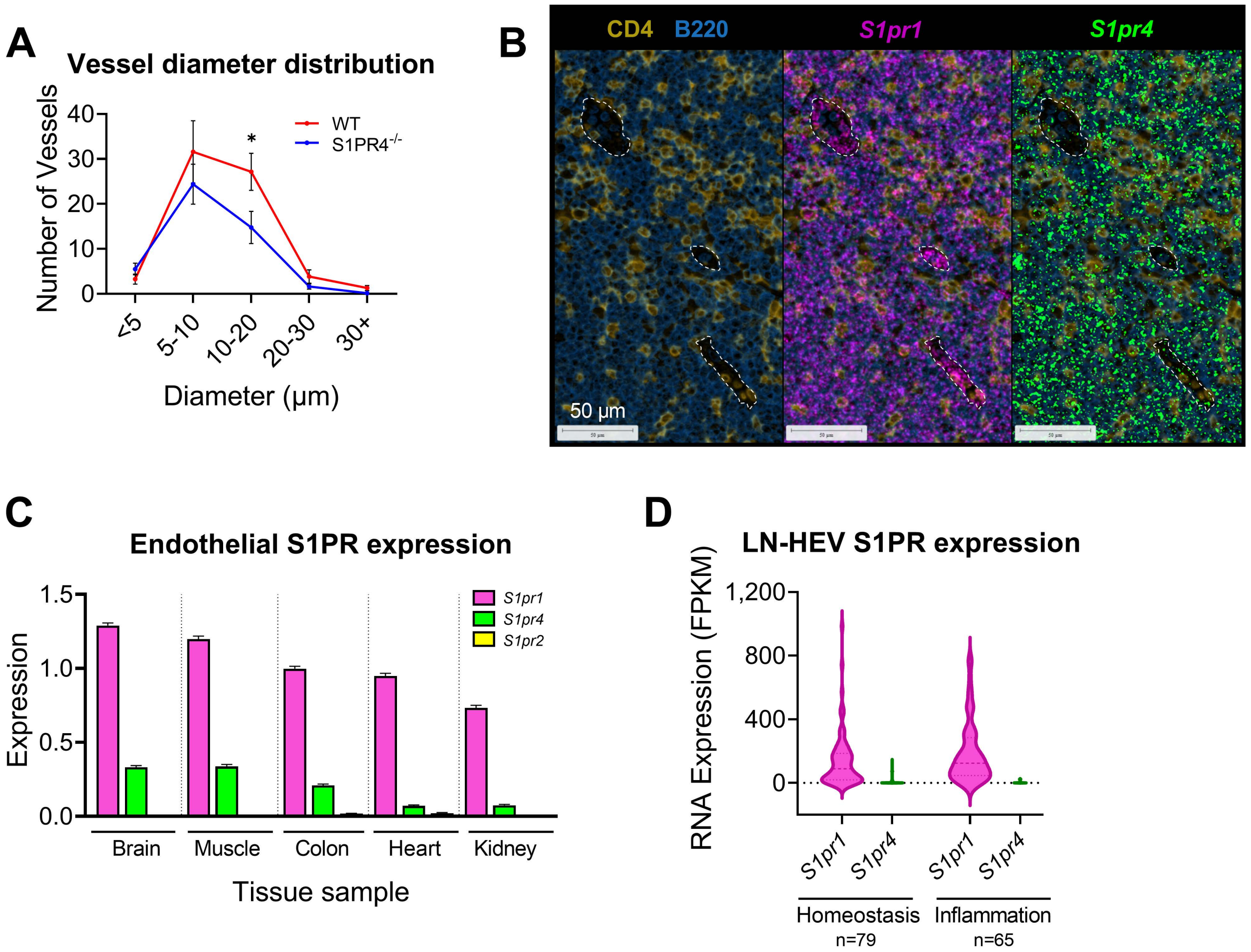

Supplemental Figure 8. Vessel diameter distribution in dLN of immunized WT and S1PR4$^{-/-}$ mice; and expression of S1PRs in endothelial cells and HEV. (A) dLN from WT and S1PR4$^{-/-}$ mice were collected on Day 9 post-immunization and stained with anti-CD31 to identify the vascular network. The number of identifiable CD31^{+} vessel segments were quantified and sorted according to vessel diameters. Each data point represents the binned average from one tissue section from a single mouse (n=7-8 mice). Data represent Mean ± SEM; *p<0.05 using an unpaired t-test. **(B)** Representative confocal image from a Day 9 WT dLN probed for *S1pr1* (magenta) and *S1pr4* (green) via RNAscope *in situ* hybridization and multiplexed with CD4 (tan) and B220 (blue) immunohistochemistry. White outline delineates vesssel, highlighting the presence of *S1pr1* expression and absence of *S1pr4*. Scale bar, 50 µm. **(C)** Expression of *S1pr1*, *S1pr4*, and *S1pr2* mRNA in endothelial cells from various tissues, prepared using publicly available single-cell transcriptomics data from the EC Atlas (https://carmelietlab.sites.vib.be/en/software-tools). Data represent Mean ± SEM. **(D)** Violin plots showing HEV expression of *S1pr1* and *S1pr4* mRNA in homeostasis and inflammation, as extracted from a publicly accessible database from a study performing single cell RNA-Seq on these cell populations (*Single-Cell Analysis Reveals Heterogeneity of High Endothelial Venules and Different Regulation of Genes Controlling Lymphocyte Entry to Lymph Nodes*) (GEO: GSE106514).

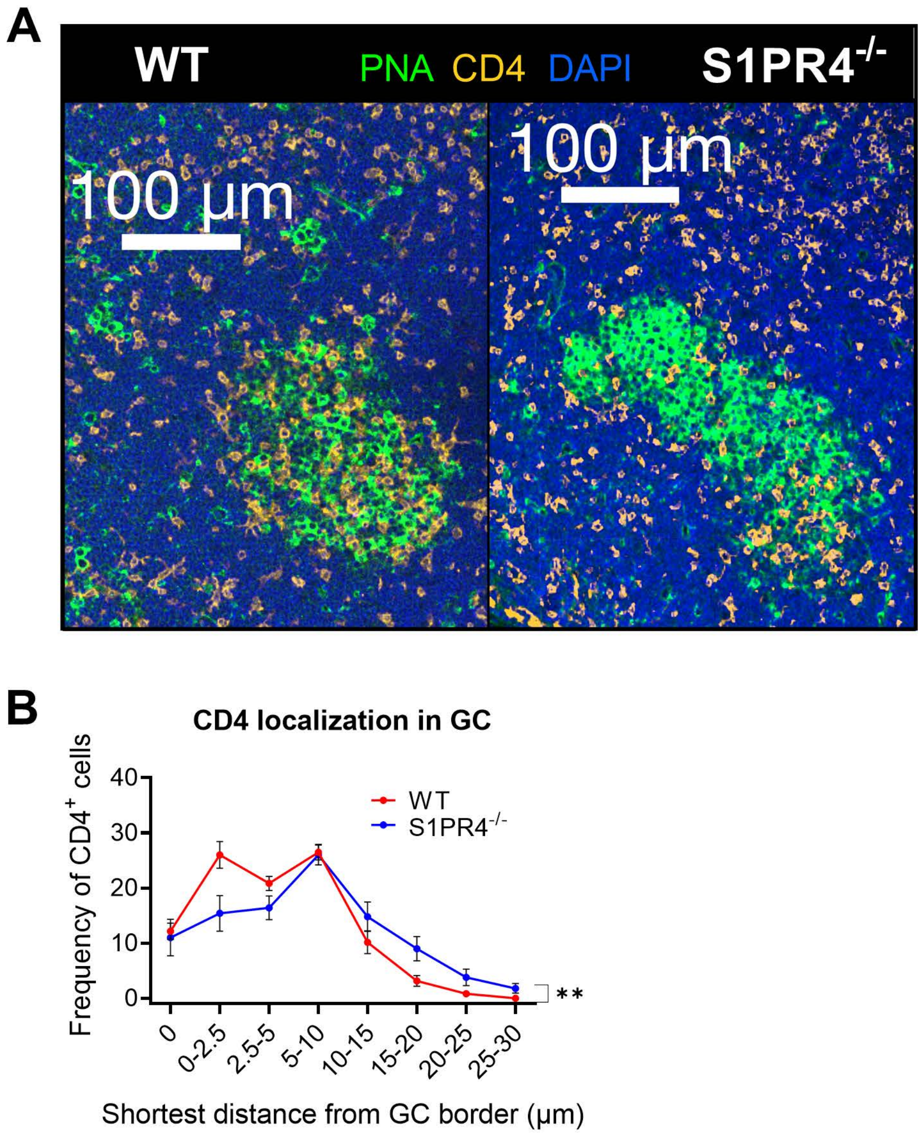

Supplemental Figure 9. Apparent differences in localization of T_{FH} cells in dLN of immunized $S1PR4^{-/-}$ mice. (A) Representative images showing $CD4^{+}$ T cell localization within GC of dLN 9 days post-immunization. GC B cells identified with PNA in green, T cells stained with anti-CD4 in yellow, and cell nuclei stained with DAPI in blue. Visual differences in localization between WT (left) and $S1PR4^{-/-}$ (right) were quantified using Imaris software to determine **(B)** the shortest distance of $CD4^{+}$ T cells to the border of the GC. Distances were normalized by the area of the GC, and frequency of T_{FH} cells at the indicated distance intervals was calculated using an Excel function. Each data point includes the analysis of all the GCs in each LN section (6 WT and 5 $S1PR4^{-/-}$ from individual mice). The combined total number of GC analyzed was 31 for WT and 22 for $S1PR4^{-/-}$. Data represent Mean ± SEM; **$p<0.01$ between curves using a two-way ANOVA.
