# Supplementary material for: Sphingosine-1-Phosphate Receptor 4 links neutrophils and early local inflammation to lymphocyte recruitment into the draining lymph node to facilitate robust germinal center formation
Source: Front Immunol. 2024 Aug 12;15:1427509. doi: 10.3389/fimmu.2024.1427509 (PMC11345157; doi:10.3389/fimmu.2024.1427509)
Supplement: Supplementary file 2 [file Table_1.docx]

| **Target** | **Dilution** | **Clone** | **Vendor** |
| --- | --- | --- | --- |
| *Immunofluorescence* |  |  |  |
| Anti-mouse PNAd, AF 647 | 1:100 | MECA79 | Biolegend |
| Anti-mouse CD31, PE | 1:100 | 390 | Biolegend |
| Anti-mouse IgD, AF 700 | 1:100 | 11-26c.2a | Biolegend |
| Anti-mouse GL7 Antigen, AF-488 | 1:400 | GL7 | Biolegend |
| Anti-mouse CD4 | 1:200 | GK1.5 | Biolegend |
| *Immunohistochemistry* |  |  |  |
| Purified anti-mouse CD4 | 1:50 | 4SM95 | eBioscience |
| Purified anti-mouse CD45R/B220 | 1:20 | RA3-6B2 | BD Biosciences |
| Peanut agglutinin (PNA), biotin | 1:100 | -- | Vector Labs |
| Streptavidin, AF 488 | 1:100 | -- | Invitrogen |
| AffiniPure Goat anti-HRP, AF 647 | 1:50 | -- | Jackson Immuno Research Labs |
| *RNAscope multiplex* |  |  |  |
| Anti-mouse CD4, biotin | 1:250 | 13-9766-82 | eBioscience |
| Anti-mouse CD45R/B220, biotin | 1:400 | RA3-6B2 | BD Biosciences |

**Supplemental Table 1. Staining reagents used in immunofluorescence and immunohistochemistry**
