# Supplementary material for: Sphingosine-1-Phosphate Receptor 4 links neutrophils and early local inflammation to lymphocyte recruitment into the draining lymph node to facilitate robust germinal center formation
Source: Front Immunol. 2024 Aug 12;15:1427509. doi: 10.3389/fimmu.2024.1427509 (PMC11345157; doi:10.3389/fimmu.2024.1427509)
Supplement: Supplementary file 3 [file Table_2.docx]

| **Target** | **Clone** | **Vendor** |
| --- | --- | --- |
| *B cells (CD19^+^ or B220^+^, TCR*β*^-^)* |  |  |
| Anti-mouse CD19, BV 786 | 6D5 | Biolegend |
| Anti-mouse B220, BV 786 | RA3-6B2 | BD Biosciences |
| *GC B cells (GL7^+^ Fas^+^)* |  |  |
| Anti-mouse GL7, AF 488 | GL7 | Biolegend |
| Anti-mouse Fas/CD95, PE/Cy7 | Jo2 | BD Biosciences |
| *T cells (TCR*β*^+^, CD19^-^ or B220^-^)* |  |  |
| Anti-mouse TCRβ, BV 605 | H57-597 | Biolegend |
| *T_FH_ cells (CD4^+^ PD1^+^ CXCR5^+^)* |  |  |
| Anti-mouse CD4, BUV 737 | GK1.5 | BD Biosciences |
| Anti-mouse PD1, BV 421 | RMP1-30 | BD Biosciences |
| Anti-mouse CXCR5, APC | L138D7 | Biolegend |
| *Neutrophils (CD11b^+^ Ly6G^+^)* |  |  |
| Anti-mouse CD11b, BUV 395 | M1/70 | BD Biosciences |
| Anti-mouse Ly6G, PE/Cy7 | 1A8 | Biolegend |
| *Monocytes (CD11b^+^ Ly6C^+^)* |  |  |
| Anti-mouse Ly6C, AF 488 | HK1.4 | Biolegend |
| *Dendritic cells (CD11c^+^ MHC II^+^)* |  |  |
| Anti-mouse CD11c, PE | N418 | Biolegend |
| Anti-mouse I-A/I-E, AF 700 | M5/114.15.2 | Biolegend |
| *B cell antibody production* |  |  |
| Anti-mouse IgG1, UNLB or BV 421 | RMG1-1 | Biolegend |
| Anti-mouse IgG2b, UNLB or APC | RMG2b-1 | Biolegend |
| Anti-mouse IgG3, FITC | R40-82 | BD Biosciences |
| Anti-mouse IgG3, UNLB | R2-38 | BD Biosciences |
| Anti-mouse IgE, UNLB or FITC | RME-1 | Biolegend |
| *T cell cytokine production* |  |  |
| Anti-mouse IFNy, AF 488 | XMG1.2 | Biolegend |
| Anti-mouse IL-4, PE | 11B11 | Biolegend |
| *T cell transcription factors* |  |  |
| Anti-mouse Tbet, AF 488 | 4B10 | Biolegend |
| Anti-mouse GATA3, AF 647 | 16E10A23 | Biolegend |
| *Lymphocyte proliferation* |  |  |
| Anti-mouse Ki67, BUV 395 | B56 | BD Biosciences |

**Table 2. Antibodies used for population identification by flow cytometry.**
