# Supplementary material for: Sphingosine-1-Phosphate Receptor 4 links neutrophils and early local inflammation to lymphocyte recruitment into the draining lymph node to facilitate robust germinal center formation
Source: Front Immunol. 2024 Aug 12;15:1427509. doi: 10.3389/fimmu.2024.1427509 (PMC11345157; doi:10.3389/fimmu.2024.1427509)
Supplement: Supplementary file 5 [file Table_4.docx]

**Supplemental Table 4. Immune cell populations and characteristics in naive WT and S1PR4^-/-^ tissues.**

|  | | **WT** | | **S1PR4^-/-^** | | ***n*** | ***p*-value** | |
| --- | --- | --- | --- | --- | --- | --- | --- | --- |
|  | | Percent (%) | Total (10^-3^) | Percent (%) | Total (10^-3^) |  | Percent | Total |
| **Blood**  **(100 µL)** | Cellularity | -- | 176.4 ± 12.7 | -- | 164.4 ± 12.2 | 23 | -- | 0.50 |
|  | % HCT | 44.7 ± 0.9 | -- | 43.8 ± 1.4 | -- | 6 | 0.60 | -- |
|  | B cells | 29.6 ± 1.9 | 48.1 ± 4.8 | 28.6 ± 1.8 | 46.8 ± 4.8 | 24 | 0.69 | 0.85 |
|  | T cells | 27.8 ± 1.5 | 47.2 ± 4.0 | 29.3 ± 1.6 | 47.8 ± 4.5 | 22 | 0.53 | 0.93 |
|  | CD4+ | 68.2 ± 1.6 | 32.8 ± 2.8 | 66.2 ± 1.7 | 32.3 ± 3.2 | 20 | 0.41 | 0.91 |
|  | CD8+ | 30.9 ± 1.4 | 32.0 ± 3.5 | 33.0 ± 1.6 | 31.7 ± 4.1 | 20 | 0.34 | 0.96 |
|  | Neutrophils | 12.6 ± 1.0 | 24.3 ± 3.1 | 11.8 ± 0.8 | 21.2 ± 2.3 | 26 | 0.54 | 0.42 |
|  | Monocytes | 3.3 ± 0.3 | 5.7 ± 1.0 | 3.2 ± 0.2 | 5.1 ± 0.5 | 14 | 0.71 | 0.60 |
|  | | Percent (%) | Total (10^-3^) | Percent (%) | Total (10^-3^) |  | Percent | Total |
| **Spleen** | Cellularity | -- |  | -- | 40.07 | 23 | -- | 0.45 |
|  | Weight (mg) | -- | 74.8 ± 2.5 | -- | 72.3 ± 2.2 | 24 | -- | 0.46 |
|  | B cells | 51.1 ± 1.3 | 24.5 ± 3.4 | 51.0 ± 1.5 | 22.1 ± 3.3 | 24 | 0.95 | 0.62 |
|  | T cells | 29.8 ± 1.1 | 15.0 ± 2.1 | 29.0 ± 1.4 | 13.3 ± 2.3 | 22 | 0.67 | 0.58 |
|  | CD4+ | 61.9 ± 1.5 | 8.6 ± 1.2 | 61.3 ± 1.7 | 8.0 ± 1.4 | 20 | 0.79 | 0.76 |
|  | CD8+ | 37.2 ± 1.5 | 5.5 ± 0.9 | 37.7 ± 1.7 | 5.0 ± 0.9 | 20 | 0.85 | 0.64 |
|  | Neutrophils | 3.1 ± 0.4 | 1.1 ± 0.1 | 3.0 ± 0.4 | 1.1 ± 0.1 | 26 | 0.86 | 0.74 |
|  | Monocytes | 1.6 ± 0.1 | 1.1 ± 0.2 | 1.4 ± 0.1 | 1.0 ± 0.1 | 14 | 0.19 | 0.36 |
|  | Dendritic cells | 1.9 ± 0.3 | 1.5 ± 0.1 | 1.6 ± 0.3 | 1.2 ± 0.1 | 7 | 0.47 | 0.03 |
|  | | Percent (%) | Total (10^-3^) | Percent (%) | Total (10^-3^) |  | Percent | Total |
| **Popliteal LN** | Cellularity | -- | 414.0 ± 34.2 | -- | 416.2 ± 35.0 | 20 | -- | 0.96 |
|  | B cells | 40.1 ± 2.2 | 172.7 ± 20.5 | 39.1 ± 2.0 | 156.1 ± 14.6 | 22 | 0.74 | 0.51 |
|  | T cells | 55.2 ± 2.1 | 228.1 ± 22.1 | 56.1 ± 1.9 | 227.4 ± 24.4 | 22 | 0.77 | 0.98 |
|  | CD4+ | 54.3 ± 0.52 | 116.8 ± 11.3 | 53.0 ± 0.50 | 113.8 ± 12.0 | 21 | 0.08 | 0.86 |
|  | CD8+ | 45.3 ± 0.54 | 102.8 ± 10.5 | 46.6 ± 0.51 | 107.7 ± 12.6 | 21 | 0.09 | 0.77 |
|  | Neutrophils | 0.15 ± 0.01 | 0.73 ± 91 | 0.19 ± 0.02 | 0.92 ± 0.1 | 14 | 0.14 | 0.26 |
|  | Dendritic cells | 0.40 ± 0.03 | 2.0 ± 0.30 | 0.33 ± 0.03 | 1.7 ± 0.2 | 14 | 0.13 | 0.36 |
|  | | Percent (%) | Total (10^-3^) | Percent (%) | Total (10^-3^) |  | Percent | Total |
| **Bone Marrow** | Cellularity | -- | 13.9 ± 1.1 | -- | 13.0 ± 0.9 | 7 | -- | 0.57 |
|  | Neutrophils | 39.1 ± 3.2 | 5.6 ± 0.8 | 37.0 ± 2.5 | 4.8 ± 0.5 | 7 | 0.62 | 0.43 |
|  | Monocytes | 8.6 ± 0.5 | 1.7 ± 0.1 | 8.5 ± 0.61 | 1.1 ± 0.1 | 7 | 0.92 | 0.62 |

The frequency and absolute number of the indicated populations was analyzed by flow cytometry and reported as Mean ± SD. N indicates number of mice evaluated. p-value was determined using unpaired t-tests.
